# Establishment and validation of an interpretable machine learning-based predictive model for risk of post-PCI in-hospital heart failure in AIHD patients

**DOI:** 10.3389/fcvm.2026.1785285

**Published:** 2026-02-27

**Authors:** Xinying Zhao, Zhihang Wang, Qiqi Yang, Huiqi Liu, Yigen Li, Xi Ye

**Affiliations:** 1 The Affiliated Guangzhou Hospital of TCM of Guangzhou University of Chinese Medicine, Guangzhou, Guangdong, China; 2 School of Computer Science, University of Bristol, Bristol, United Kingdom; 3 National TCM Master Lin Tiandong's Heritage and Inheritance Studio, The Affiliated Guangzhou Hospital of TCM of Guangzhou University of Chinese Medicine, Guangzhou, Guangdong, China

**Keywords:** acute ischemic heart disease, machine learning, percutaneous coronary intervention, predictive models, risk of in-hospital heart failure

## Abstract

**Background:**

This study intends to establish and validate an interpretable machine learning (ML) model based on clinical features for early prediction of the risk of post-percutaneous coronary intervention (PCI) in-hospital heart failure (HF) in patients with acute ischemic heart disease (AIHD).

**Methods:**

This study retrospectively included AIHD patients who underwent PCI at the Affiliated Guangzhou Hospital of TCM of Guangzhou University of Chinese Medicine from January 2023 to May 2025. LASSO regression was utilized for feature screening first, and then seven predictive models for HF risk in AIHD patients were established using ML algorithms. The model performance was fully assessed on the validation set through the area under the curve (AUC) with 95% CI, calibration curve and expected calibration error, recall, F1-score, positive predictive value, negative predictive value, and accuracy, and internal validation was conducted using the Bootstrap method. In addition, feature importance was evaluated by SHapley Additive exPlanations (SHAP) values, and individualized predictions were explained by Local Interpretable Model-Agnostic Explanations (LIME).

**Results:**

Two hundred and three patients with AIHD were ultimately included, of whom 55 (27.1%) experienced in-hospital HF. Of the seven ML models, the random forest (RF) model demonstrated optimal performance on the validation set, with an AUC of 0.70 (95% CI 0.53–0.84) and an accuracy of 0.77; the calibration curve revealed high agreement between predicted and actual risks. Twelve predictive features associated with endpoint events were identified by LASSO regression, and the top five features contributing to the predictive efficacy of the RF model were age, monocyte count, heart rate, platelet count, and mean platelet volume according to the ranking of feature importance. In addition, the contribution of features to the prediction of HF risk was visualized by SHAP summary plots and LIME.Finally, an open Web-based prediction tool was deployed.

**Conclusion:**

This exploratory study developed a random forest (RF) model to predict the risk of post-PCI in-hospital HF in patients with AIHD. Based on the SHAP and LIME methods, the clinical interpretability of the model was significantly enhanced. Future research with larger sample sizes is warranted to optimize the training set and validate the generalizability of the model.

## 1 Introduction

Acute ischemic heart disease (AIHD) is a critical clinical syndrome in which acute ischemia, injury, and necrosis of cardiomyocytes are triggered due to a dramatic decline or interruption of coronary blood flow. The major clinical subtypes of AIHD include unstable angina, ST-elevation myocardial infarction, and non-ST-elevation myocardial infarction. As the most severe manifestation of coronary heart disease, AIHD has become a major public health issue, with an estimated global median age-standardized incidence of 293.3/100,000, and also one of the leading causes of death (1). If not promptly relieved, myocardial ischemia and hypoxia will progress to irreversible myocardial necrosis (i.e., myocardial infarction), causing impairment of cardiac function. According to data, even after percutaneous coronary intervention (PCI), the mortality rate within 2 years after operation can reach as high as 4.29% (2). Particularly, heart failure (HF) is an important complication, and the incidence of in-hospital HF in patients with AIHD reaches 13%–17.9% (3, 4); once HF occurs in AIHD, patients may face a significantly higher in-hospital mortality (3-fold increase), prolonged hospitalization, and decline in quality of life, with an unfavorable long-term prognosis (5).

HF originates primarily from impairment of ventricular systolic function triggered by massive myocardial ischemia and necrosis, as well as a vicious cycle of secondary events [over-activation of neuroendocrine systems [especially the renin-angiotensin-aldosterone system and the sympathetic nervous system], pathologic ventricular remodeling [ventricular dilatation and fibrosis], and persistent inflammatory response] (6–8). Moreover, underlying diseases such as hypertension, diabetes, chronic kidney disease, and chronic lung disease are independent risk factors for the development of HF. All-cause mortality in AIHD can be significantly reduced by percutaneous coronary intervention (PCI), but HF remains a major contributor to adverse clinical outcomes (9, 10). Therefore, early and accurate identification of high-risk groups for in-hospital HF in AIHD is crucial. In this way, clinicians can initiate risk-stratified management and adopt targeted myocardial protection strategies (e.g., hemodynamic optimization, inhibition of neuroendocrine activation) and strengthened secondary prevention, thereby maximizing the myocardial protective effect and delaying the deterioration of cardiac function. Ultimately, the all-cause mortality decreases, and both long-term quality of life and prognosis are ameliorated (11).

Integrating multi-source clinical data (e.g., demographics, vital signs, laboratory indicators, electrocardiograms, and imaging findings), machine learning (ML) models have exhibited great potential in predicting the onset, progression, and prognosis of heart diseases, which can capture complex nonlinear relationships of variables to more accurately assess individualized risks (12, 13). For example, Lin et al. developed an ML model for predicting the risk of HF within 3 years following acute myocardial infarction (14). However, predicting the risk of in-hospital HF in AIHD can more directly benefit real-time clinical decision-making (e.g., selection of intensity of monitoring, adjustment of interventions, and resource allocation). Therefore, this study intends to establish an ML model for predicting the risk of in-hospital HF in AIHD. To enhance the model's clinical applicability and acceptability, its interpretability was improved by SHapley Additive exPlanations (SHAP) values (15). SHAP can quantify the contribution of features to the prediction, and reveal the complex relationship of features with the outcome, transparentizing the decision-making process of the “black-box” model (16). Using SHAP, clinicians can understand the basis for the prediction and implement individualized risk-based interventions, achieving early identification and precision prevention.

In this study, seven ML models for predicting the risk of post-PCI in-hospital HF in AIHD patients were established based on clinical features and validated, and the prediction mechanism was visualized by SHAP and LIME techniques, greatly enhancing the model's clinical applicability.

## 2 Methods

### 2.1 Study design

This retrospective modeling study covered modeling, validation, and interpretation. First, the predictive models were preliminarily developed using seven ML algorithms. Then the model performance was fully assessed on the validation set through the area under the curve (AUC) with 95% CI, calibration curve and expected calibration error (ECE), recall, F1-score, positive predictive value (PPV), negative predictive value (NPV), and accuracy, based on which the optimal model was identified. Finally, the optimal model's prediction mechanism and key features were explanatorily analyzed using SHAP and LIME techniques, and an open Web-based prediction tool was deployed.

### 2.2 Data source and sample size estimation

Data were acquired using electronic medical records from a retrospective cohort of AIHD patients who underwent PCI at the Affiliated Guangzhou Hospital of TCM of Guangzhou University of Chinese Medicine from January 2023 to May 2025. This study was approved by the Institutional Review Board of the hospital (No. 2024NK60), and informed consent was waived because of its retrospective nature.

Following the 10 EPV principle, the minimum number of positive events required for the training set was estimated based on the number of predictor variables included (17).

### 2.3 Participants and outcome definitions

AIHD patients who underwent PCI at the Affiliated Guangzhou Hospital of TCM of Guangzhou University of Chinese Medicine from January 2023 to May 2025 were included. Inclusion criteria: (a) patients aged >18 years; and (b) patients meeting the diagnostic criteria for AIHD in the current guidelines (18), with clinical symptoms, characteristic electrocardiographic changes, and elevated levels of cardiac biomarkers at the same time, specifically as follows: (i) ST-segment elevation myocardial infarction (STEMI): cardiac troponin (cTn) > 99th percentile upper limit of normal (ULN) or creatine kinase-myocardial band (CK-MB) > 99th percentile ULN, with electrocardiogram (ECG) showing upward convex ST-segment elevation, accompanied by one or more of persistent ischemic chest pain, echocardiogram showing segmental wall motion abnormalities, and coronary angiogram showing abnormalities; (ii) Non-ST-segment elevation myocardial infarction: cTn >99th percentile ULN or CK-MB > 99th percentile ULN, accompanied by one or more of persistent ischemic chest pain, ECG showing new ST-segment depression or flattened/inverted T waves, echocardiogram showing segmental wall motion abnormalities, and coronary angiogram showing abnormalities; (iii) Unstable angina (UA): negative cTn, ischemic chest pain, ECG showing transient ST-segment depression or flattened/inverted T waves, rarely with ST-segment elevation (vasospastic angina). Exclusion criteria: (a) patients with a history of HF; (b) patients who died during hospitalization or with a length of stay <48 h; (c) patients who experienced major clinical events recently (in the last month): major surgery, severe trauma, shock, active infections, or systemic inflammation; (d) patients with severe immune dysfunction; and (e) patients with active malignancy or hematologic malignancy.

The primary outcome of this study was the incidence of in-hospital HF in patients following PCI. The diagnosis of HF was based on the 2022 American College of Cardiology, American Heart Association, and Heart Failure Society of America Guidelines for the Management of Heart Failure (19), specifically as follows: (1) Clinical symptoms and signs: typical symptoms include dyspnea at rest or during physical activity, orthopnea, paroxysmal nocturnal dyspnea, fatigue, and decreased exercise tolerance. Signs include pulmonary crackles, peripheral edema, distension of jugular vein, and positive hepatojugular reflux. (2) Cardiac imaging (primarily echocardiography) revealing structural or functional abnormalities of the heart [e.g., reduced left ventricular ejection fraction (LVEF), cardiac chamber enlargement]. (3) Natriuretic peptide levels: B-type natriuretic peptide (BNP) > 35 pg/mL or N-terminal proB-type natriuretic peptide (NT-proBNP) > 125 pg/mL.

### 2.4 Feature selection

Based on the available literature and clinical practice, 118 features related to the risk of HF were extracted from the electronic medical records, covering demographics, comorbidities, vital signs, laboratory indicators, and angiography findings. They were collected within 24 h post-admission or during PCI. Each variable was independently checked by two researchers and confirmed to have been collected at a time point earlier than the first in-hospital diagnosis of HF for the corresponding patient before it was included in the model analysis. A complete list of features is shown in Supplementary Table S1.

### 2.5 Statistical analysis

Categorical variables were described by frequencies (percentages), and compared between groups by the chi-square test or Fisher's exact test. Continuous variables of normal distribution were presented as mea*n* ± standard deviation, and compared between groups by the independent-samples *t*-test; continuous variables of non-normal distribution were presented as median (IQR), and compared between groups by the Mann–Whitney *U*-test. In the handling of outliers, extreme values were adjusted using two-sided 1% Winsorization (capping method) based on clinical significance and statistical distribution characteristics. Specifically, observations below the 1st percentile were raised to the 1st percentile level, while those above the 99th percentile were lowered to the 99th percentile level. This approach was designed to preserve the potential biological significance of extreme values while effectively reducing their excessive influence on the statistical model. Features with less than 30% missing values were processed by employing the Multiple Imputation by Chained Equations (MICE) method with the mice package in R. To adapt the model to potential nonlinear relationships, a random forest approach was adopted for imputation. Five imputed datasets (m = 5) were generated with a fixed random seed (123) to ensure reproducibility of the results. Features were then screened using L1 regularization based on LASSO regression. The optimal regularization parameter (*λ*_min) was determined through cross-validation, and key predictive features were selected based on this parameter. Subsequently, for the selected features, Pearson correlation coefficients were used to assess multicollinearity among the variables. Features with high multicollinearity (|r| ≥ 0.8) were excluded to ensure unbiased estimation of model parameters. After the above preprocessing, the retained predictor variables were utilized to establish the predictive model.

Then the samples were randomized into a training set (70%) and a testing set (30%). Seven ML algorithms were used: SVM, KNN, XGBoost, DT, NB, RF, and LR. The models were trained on the training set, and the model performance was assessed on the validation set from the discrimination (AUC with 95% CI), calibration (calibration curve and ECE), and classification (accuracy, recall, F1-score, PPV, and NPV). Finally, based on the assessment results, the optimal model was selected. To thoroughly evaluate the robustness of the stochastic optimal model and mitigate potential bias arising from the limited sample size, the Bootstrap method was employed for internal validation. By performing 1,000 Bootstrap resampling iterations on the dataset, the model was trained using Bootstrap samples in each iteration. Model performance was comprehensively evaluated by calculating the average area under the curve (AUC) and its 95% CI, ECE, recall, F1-score, PPV, and NPV based on out-of-bag (OOB) samples.

In addition, the model's interpretability was enhanced using the SHAP technique. First, we displayed the overall contribution of features to the prediction using SHAP summary plots, and analyzed the relation between individual features and predictions using SHAP dependence plots. Finally, the contribution of important features to the individual prediction was visualized using SHAP force plots and LIME.To operationalize the model, the best-performing algorithm was packaged into an interactive web application using Python's Streamlit framework; this deployment serves both to validate the model's reproducibility in a practical environment and to provide an accessible interface for instant, on-demand predictions.

R 4.4.3 and Python 3.12.7 were utilized for data analysis, modeling, and validation. Statistical significance was assumed at *P* < 0.05.

## 3 Results

### 3.1 Participants

**Figure 1:**
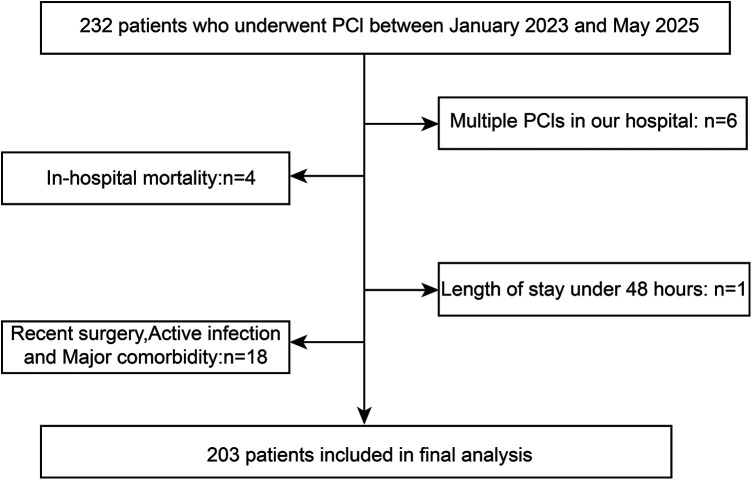
Patient selection flowchart.

Based on the eligibility criteria, 232 AIHD patients were initially screened, of whom six were excluded due to multiple PCI procedures in our hospital, four were excluded due to in-hospital death, one was excluded due to a length of stay <48 h, and 18 were excluded due to a history of surgical procedures, severe infections, and major illnesses. Ultimately, the study cohort encompassed 203 patients (Figure 1).

Following the 10 EPV principle, 12 predictor variables were included: age, AST/ALT, cystatin C, myoglobin, number of diseased vessels, heart rate (HR), absolute neutrophil count, direct bilirubin, lactate dehydrogenase (LDH), mean platelet volume (MPV), platelet count (PLT), and presence or absence of triple vessel disease, with HF as the primary outcome metric. Of these variables, continuous indicators (e.g., age, HR, and myoglobin) each corresponded to one *β* coefficient, and the dichotomous indicator (presence or absence of triple vessel disease) corresponded to one *β* coefficient. Therefore, twelve *β* coefficients were involved in the model, and at least 120 positive outcome events were required for constructing the training set. However, only 40 (<120) positive outcome events were involved in the 142 training samples in this study, suggesting an insufficient sample size.

All patients were assigned to an HF group (*n* = 55) and a non-HF group (*n* = 148). The HF group had a significantly higher age of patients (*P* < 0.001) and a significantly lower proportion of smokers (*P* = 0.035) than the non-HF group. In the HF group, significant hemodynamic deterioration occurred: elevation of HR (*P* = 0.005), and significant decreases in systolic and diastolic blood pressure (*P* < 0.05); coronary artery lesions became significantly more severe: a higher proportion of triple vessel disease (*P* = 0.019), a higher prevalence of left main coronary artery lesions (*P* = 0.004), a significant aggravation of stenosis (percentage of reduction of lumen diameter) in the proximal (*P* = 0.006) and middle (*P* = 0.001) segments of the anterior descending branch, and a significant difference in the distribution of culprit vessels (*P* = 0.013). Moreover, the levels of markers of myocardial injury significantly rose in the HF group, and significant inflammatory responses also occurred, as manifested by elevation of neutrophil count, monocyte count (MONO), and D-dimer, accompanied by a decreased lymphocyte percentage. In addition, the HF group exhibited more severe renal impairment and hepatic dysfunction, as well as electrolyte disturbance (hypocalcemia and hyperphosphatemia) (Table 1).

**Table 1:** Baseline demographic and clinical characteristics of 203 patients with acute ischemic heart disease post-PCI.

Characteristics	level	Non-Heart failure(*n* = 148)	Heart failure(*n* = 55)	*p*-value
Demographic characteristics				
Gender, *n* (%)	Female	28 (18.9)	14 (25.5)	0.408
	Male	120 (81.1)	41 (74.5)	
Age, year		62.00 [53.00, 70.00]	71.00 [62.00, 80.00]	<0.001
Vital signs				
Heart rate, bmp		75.00 [70.00, 85.00]	85.00 [73.00, 94.00]	0.005
Systolic blood pressure, mmHg		135.00 [121.00, 151.25]	127.00 [119.00, 139.00]	0.048
Diastolic blood pressure, mmHg		80.00 [73.00, 89.00]	76.00 [67.00, 81.50]	0.003
Hypertension, *n* (%)	Yes	76 (51.4)	30 (54.5)	0.805
	No	72 (48.6)	25 (45.5)	
Diabetes, *n* (%)	Yes	41 (27.7)	21 (38.2)	0.204
	No	107 (72.3)	34 (61.8)	
Coronary heart disease, *n* (%)	Yes	20 (13.5)	6 (10.9)	0.797
	No	128 (86.5)	49 (89.1)	
Hyperlipidemia, *n* (%)	Yes	20 (13.5)	7 (12.7)	1.000
	No	128 (86.5)	48 (87.3)	
Smoking, *n* (%)	Yes	72 (48.6)	17 (30.9)	0.035
	No	76 (51.4)	38 (69.1)	
Heavy Drinking, *n* (%)	Yes	24 (16.2)	5 (9.1)	0.287
	No	124 (83.8)	50 (90.9)	
Previously implanted stent, *n* (%)	Yes	12 (8.1)	5 (9.1)	1.000
	No	136 (91.9)	50 (90.9)	
Classification of illness				
STEMI, *n* (%)	Yes	64 (43.2)	31 (56.4)	0.132
	No	84 (56.8)	24 (43.6)	
Coronary angiogram findings				
LM, *n* (%)	Yes	8 (5.4)	11 (20.0)	0.004
	No	140 (94.6)	44 (80.0)	
LAD, *n* (%)	Yes	137 (92.6)	53 (96.4)	0.510
	No	11 (7.4)	2 (3.6)	
pLAD (%)		40.00 [0.00, 85.00]	60.00 [30.00, 100.00]	0.006
mLAD (%)		50.00 [0.00, 85.00]	85.00 [45.00, 95.00]	0.001
dLAD (%)		0.00 [0.00, 42.50]	0.00 [0.00, 80.00]	0.298
LCX, *n* (%)	Yes	102 (68.9)	45 (81.8)	0.099
	No	46 (31.1)	10 (18.2)	
pLCX (%)		0.00 [0.00, 40.00]	0.00 [0.00, 55.00]	0.064
dLCX (%)		0.00 [0.00, 0.00]	0.00 [0.00, 20.00]	0.933
RCA, *n* (%)	Yes	118 (79.7)	46 (83.6)	0.669
	No	30 (20.3)	9 (16.4)	
pRCA(%)		0.00 [0.00, 52.50]	20.00 [0.00, 72.50]	0.684
mRCA (%)		30.00 [0.00, 71.25]	50.00 [0.00, 80.00]	0.523
dRCA (%)		0.00 [0.00, 60.00]	0.00 [0.00, 50.00]	0.996
Triple vessel lesion, *n* (%)	Yes	76 (51.4)	39 (70.9)	0.019
	No	72 (48.6)	16 (29.1)	
Main criminal vessel, *n* (%)	N/A	9 (6.1)	2 (3.6)	0.013
	LM	0 (0.0)	1 (1.8)	
	LAD	62 (41.9)	36 (65.5)	
	LCX	18 (12.2)	5 (9.1)	
	RCA	59 (39.9)	11 (20.0)	
Number of stents, *n* (%)		1.00 [1.00, 2.00]	1.00 [1.00, 2.00]	0.188
Number of criminal vessel, *n* (%)		3.00 [2.00, 3.00]	3.00 [2.50, 3.00]	0.004
Length of stents, *n* (%)		27.00 [18.00, 44.00]	29.00 [18.00, 48.50]	0.565
Laboratory findings				
Potassium, mmol/L		4.00 [3.70, 4.30]	4.00 [3.80, 4.25]	0.555
Sodium, mmol/L		140.10 [138.70, 142.02]	140.00 [138.00, 141.70]	0.213
Chloride, mmol/L		105.00 [102.40, 107.00]	103.90 [101.10, 106.15]	0.071
Bicarbonate, mmol/L		23.45 [21.08, 25.13]	23.50 [21.20, 25.50]	0.786
Calcium, mmol/L		2.28 [2.17, 2.37]	2.21 [2.15, 2.32]	0.012
Magnesium, mmol/L		0.83 [0.79, 0.88]	0.82 [0.78, 0.90]	0.581
Phosphate, mmol/L		1.05 [0.89, 1.20]	1.13 [0.98, 1.33]	0.021
Alanine Aminotransferase, U/L		24.30 [17.85, 38.50]	27.20 [15.40, 51.45]	0.898
Aspartate Aminotransferase, U/L		29.95 [21.15, 47.25]	44.40 [24.10, 110.40]	0.031
Aspartate Aminotransferase to Alanine Aminotransferase Ratio		1.32 [0.95, 2.01]	1.88 [1.23, 3.05]	0.004
Gamma-Glutamyl Transferase, U/L		30.85 [20.00, 52.25]	27.00 [17.50, 48.50]	0.321
Alkaline Phosphatase, U/L		71.50 [58.75, 91.00]	71.00 [59.00, 86.60]	0.912
Glutathione Reductase, U/L		50.00 [42.08, 59.33]	57.80 [45.65, 65.40]	0.009
*α*-L-Fucosidase, U/L		29.90 [23.48, 34.55]	28.00 [20.95, 33.05]	0.173
Total Protein, g/L		67.95 [63.40, 71.30]	65.80 [61.70, 71.10]	0.227
Albumin, g/L		39.65 [35.77, 42.15]	35.50 [33.15, 40.45]	0.002
Globulin, g/L		27.70 [24.45, 31.13]	28.00 [26.25, 33.50]	0.323
Albumin to Globulin Ratio		1.44 [1.23, 1.64]	1.22 [1.08, 1.51]	0.002
Prealbumin, mg/L		259.30 [217.07, 292.70]	224.90 [199.35, 267.10]	0.010
Total Bilirubin, μmol/L		7.55 [5.60, 10.72]	7.69 [6.15, 11.10]	0.352
Direct Bilirubin, μmol/L		3.00 [2.20, 4.00]	3.50 [2.65, 4.88]	0.016
Indirect Bilirubin, μmol/L		4.50 [2.98, 7.00]	4.40 [3.00, 6.20]	0.747
Total Bile Acids, μmol/L		4.50 [3.09, 8.00]	4.10 [2.79, 6.00]	0.376
Glucose, mmol/L		7.90 [6.24, 10.76]	7.80 [6.54, 10.99]	0.787
Uric Acid, μmol/L		365.70 [299.25, 433.80]	414.00 [326.50, 500.20]	0.047
Blood Urea Nitrogen, mmol/L		5.20 [4.42, 7.23]	7.20 [5.20, 9.26]	<0.001
Creatinine, μmol/L		80.50 [69.00, 100.25]	88.00 [76.15, 142.00]	0.010
Cystatin C, mg/L		1.04 [0.89, 1.45]	1.23 [0.99, 2.08]	0.015
Lactate Dehydrogenase, U/L		222.50 [181.15, 294.18]	295.90 [225.95, 601.50]	<0.001
Creatine Kinase, U/L		186.00 [95.75, 402.50]	238.00 [103.50, 726.50]	0.162
Creatine Kinase-Myocardial Band, U/L		14.57 [3.36, 46.75]	19.68 [5.27, 77.43]	0.144
Hydroxybutyrate Dehydrogenase, U/L		164.10 [131.83, 285.00]	221.00 [162.20, 463.80]	0.001
Total Cholesterol, mmol/L		4.64 [3.94, 5.38]	4.93 [3.93, 5.70]	0.360
Triglycerides, mmol/L		1.46 [1.05, 2.27]	1.24 [0.82, 1.94]	0.031
High-Density Lipoprotein Cholesterol, mmol/L		1.04 [0.90, 1.22]	1.14 [0.90, 1.35]	0.232
Low-Density Lipoprotein Cholesterol, mmol/L		3.08 [2.40, 3.75]	3.11 [2.66, 3.76]	0.401
Prothrombin Time, s		11.75 [11.30, 12.43]	11.80 [10.97, 12.40]	0.965
Prothrombin Time Percentage, %		98.15 [91.00, 103.95]	97.00 [89.30, 105.25]	0.488
Prothrombin Time Ratio		0.98 [0.95, 1.03]	0.99 [0.93, 1.04]	0.632
International Normalized Ratio		0.98 [0.94, 1.04]	0.99 [0.93, 1.04]	0.713
Activated Partial Thromboplastin Time, s		25.65 [23.78, 28.10]	26.80 [24.25, 28.80]	0.253
D-Dimer, mg/L FEU		0.39 [0.25, 0.65]	0.54 [0.36, 0.94]	0.014
White Blood Cell Count, ×10⁹/L		9.49 [7.76, 11.95]	10.34 [8.18, 12.54]	0.372
Neutrophil Count, ×10⁹/L		6.36 [4.92, 8.77]	7.32 [5.90, 10.53]	0.031
Neutrophil Percentage, %		71.45 [62.55, 79.40]	76.10 [65.80, 82.90]	0.153
Lymphocyte Count, ×10⁹/L		1.95 [1.25, 2.49]	1.66 [1.20, 2.34]	0.154
Lymphocyte Percentage, %		21.35 [15.17, 27.38]	15.30 [9.75, 23.20]	0.001
Monocyte Count, ×10⁹/L		0.58 [0.39, 0.78]	0.68 [0.52, 0.99]	0.007
Monocyte Percentage, %		6.45 [4.88, 8.00]	6.70 [5.30, 8.85]	0.233
Eosinophil Count, ×10⁹/L		0.09 [0.05, 0.19]	0.06 [0.01, 0.15]	0.016
Eosinophil Percentage, %		1.10 [0.40, 2.40]	0.40 [0.05, 1.95]	0.007
Basophil Count, ×10⁹/L		0.03 [0.02, 0.04]	0.03 [0.02, 0.04]	0.992
Basophil Percentage, %		0.30 [0.20, 0.40]	0.30 [0.10, 0.40]	0.440
Red Blood Cell Count, ×10^12^/L		4.56 [4.25, 5.10]	4.40 [4.06, 4.92]	0.156
Hematocrit, %		40.65 [38.05, 44.42]	39.30 [36.85, 42.85]	0.145
Mean Corpuscular Volume, fL		88.30 [85.70, 91.70]	88.90 [86.80, 91.30]	0.559
Mean Corpuscular Hemoglobin, pg		30.15 [28.87, 31.30]	30.20 [29.30, 30.85]	0.998
Mean Corpuscular Hemoglobin Concentration, g/L		339.00 [332.75, 346.00]	338.00 [332.50, 343.50]	0.409
Red Cell Distribution Width - Coefficient of Variation, %		12.90 [12.47, 13.60]	13.40 [12.50, 13.90]	0.113
Red Cell Distribution Width - Standard Deviation, fL		41.75 [39.58, 44.30]	43.20 [40.60, 46.05]	0.022
Platelet Count, ×10⁹/L		240.00 [201.50, 282.25]	214.00 [177.00, 266.00]	0.045
Mean Platelet Volume, fL		9.85 [9.28, 10.50]	10.30 [9.65, 11.10]	0.004
Plateletcrit, %		0.24 [0.20, 0.31]	0.23 [0.19, 0.28]	0.367
Platelet Distribution Width, fL		10.70 [9.38, 12.53]	11.80 [10.20, 14.20]	0.027
High-Sensitivity Cardiac Troponin T, pg/mL		293.20 [74.44, 960.30]	838.70 [95.88, 3350.76]	0.007
Myoglobin, ng/mL		72.02 [31.84, 298.59]	129.00 [64.19, 452.90]	0.007
N-Terminal pro-B-type Natriuretic Peptide, pg/mL		306.10 [81.65, 973.55]	1715.00 [459.40, 5538.00]	<0.001

LM, left main coronary artery; LAD, left anterior descending artery; LCX, left circumflex artery; RCA, right coronary artery; p, proximal; m, mid; d, distal;N/A, No intraoperative record, or multiple culprit vessels were identified.

### 3.2 Model development and validation

We extracted 118 clinical features within 24 h post-admission and during PCI. Of these, 20 features were excluded due to a data missing rate >30% (Supplementary Table S2). Subsequently, the remaining features were handled by MICE, and features in which hemoglobin was automatically identified as perfect collinearity were excluded. Finally, 97 clinical features were included for modeling (Supplementary Table S3). To identify characteristic variables associated with the risk of in-hospital HF in AIHD, LASSO regression was conducted. The optimal regularization parameter (*λ*_min) was determined by cross-validation, based on which predictor variables were screened. Ultimately, 12 predictor variables significantly associated with prognosis were screened (Supplementary Figure S1). Spearman's correlation coefficient analysis revealed that all predictor variables exhibited coefficients below 0.8, effectively preventing multicollinearity (Supplementary Figure S2). Bases on these 12 features, seven ML models were constructed for predicting the HF risk: XGBoost, RF, NB, LR, SVM, KNN, and DT. Detailed specifications and hyperparameter settings for each ML algorithm are provided in Supplementary Table S4.

**Figure 2:**
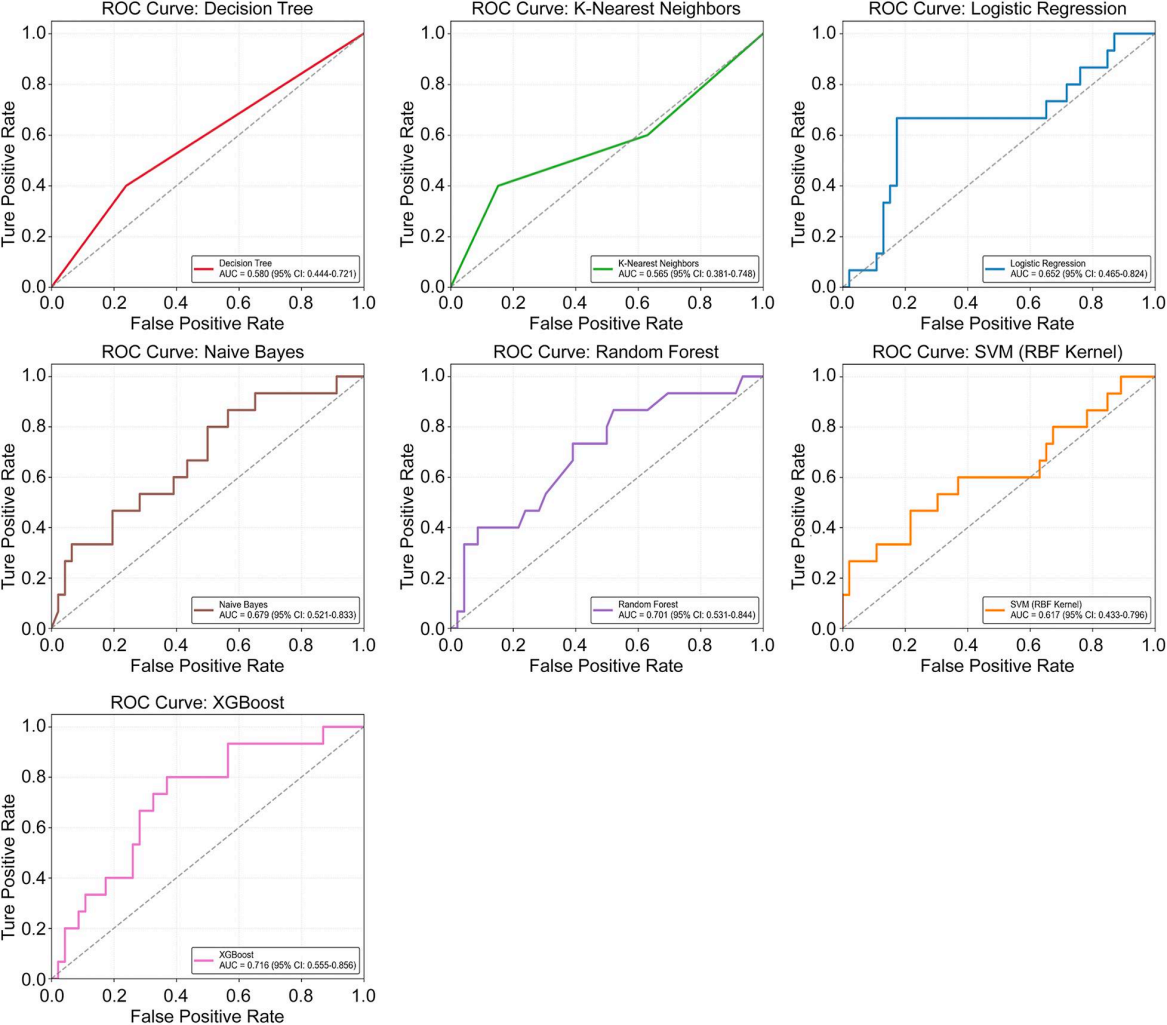
The receiver operating characteristic (ROC) plot shows how various machine learning algorithms perform on the validation dataset. AUC: Area under the curve, CI: Confidence interval, XGBoost: eXtreme gradient boosting.

**Figure 3:**
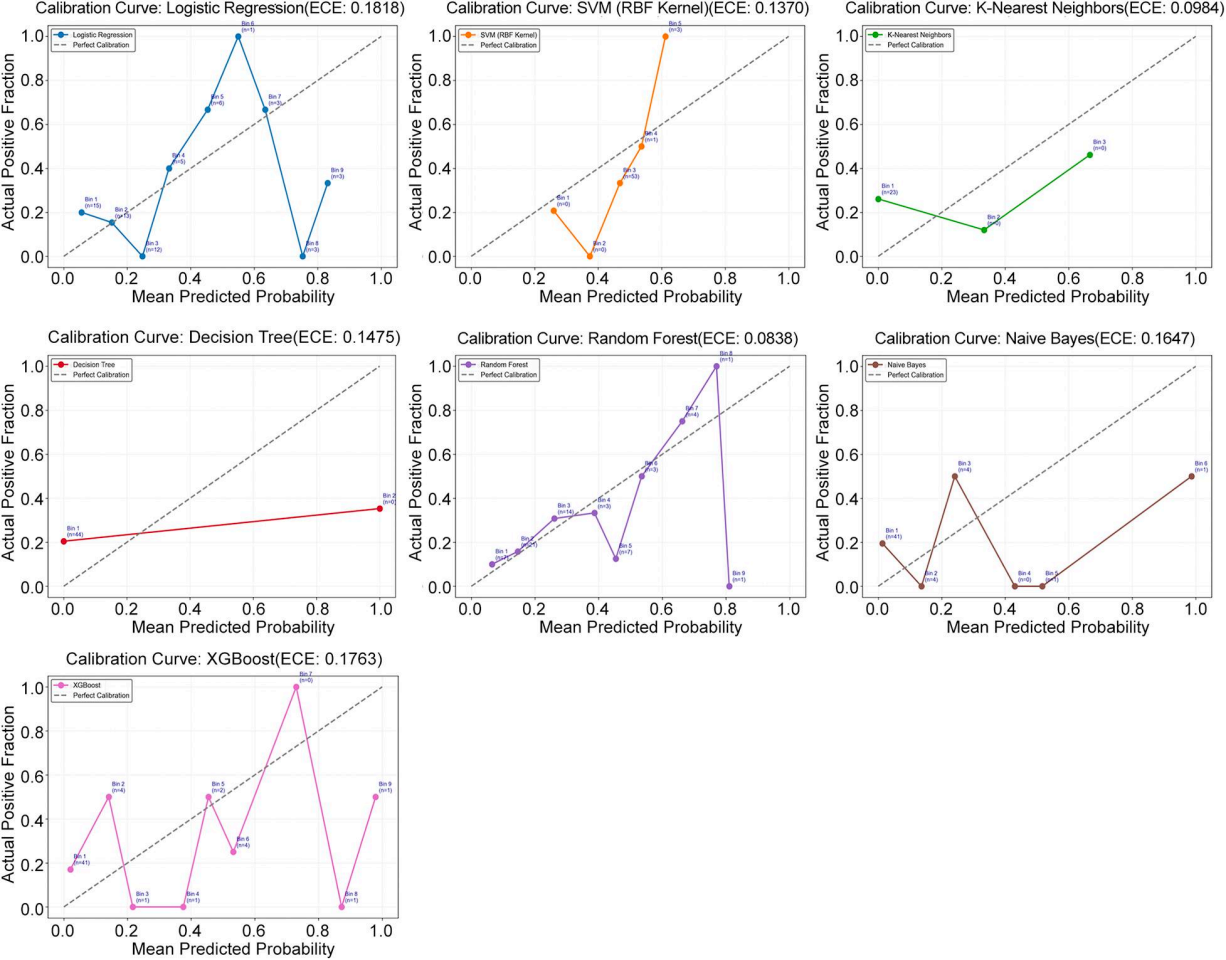
This figure shows the calibration results of several machine learning models on verification data. Each chart compares the probability of the event predicted by the model with the true probability. That diagonal represents a perfectly calibrated system, meaning that the predicted probability exactly matches the actual frequency of occurrence. The degree to which each model's curve deviates from this baseline shows how inaccurate it is in probability prediction.XGBoost:eXtreme Gradient Boosting.

**Figure 4:**
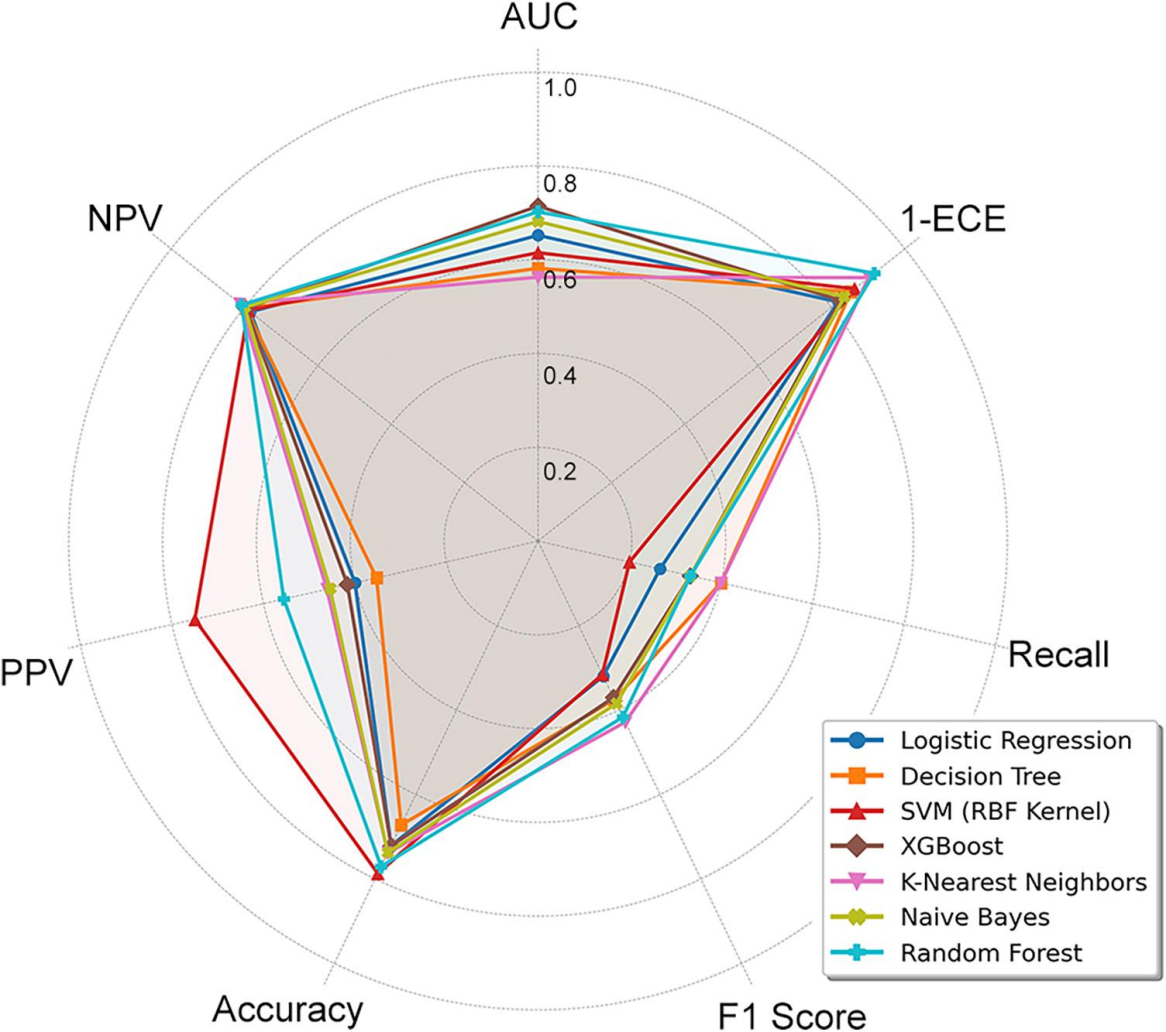
This figure evaluates seven machine learning algorithms using seven distinct criteria. The shape and size of the polygons indicate that ensemble tree-based models such as random forest (RF) yield the most stable results, whereas other models demonstrate particular strengths in specific aspects. Regarding the F1-score and 1-ECE metrics, lower values correspond to better model performance.

The AUC with 95% CI for each model is illustrated in Figure 2: Both RF (AUC 0.70; 95% CI 0.53–0.84) and XGBoost models (AUC 0.72; 95% CI 0.56–0.86) performed well in the AUC. The calibration curve and ECE for each model are illustrated in Figure 3: Both RF and NB models displayed high consistency between the predicted and actual risks, suggesting their good calibration performance. To assess the model performance more objectively, the recall, F1-score, PPV, and NPV on the validation set were further calculated and ranked. The mean rank of each model was used as its final overall rank (Supplementary Table S5), and the results were visualized by radar charts (Figure 4). To sum up, the RF model demonstrated optimal overall performance: AUC 0.70 (95% CI 0.53–0.84), ECE 0.08, Recall 0.33, F1-score 0.42, accuracy 0.77, PPV 0.56, and NPV 0.81 on the validation set. Therefore, the RF model was selected for the subsequent prediction.

To further assess the robustness of the model with a small sample size, 1,000 bootstrap replications were performed for internal validation. The validation results showed that the adjusted AUC of the model was 0.73 (95% CI: 0.63–0.83), with ECE = 0.11, recall = 0.27, F1-score = 0.35, accuracy = 0.74, PPV = 0.55, and NPV = 0.77 (Table 2). Compared to the results from the original test set, Bootstrap validation not only confirmed the discriminatory capability of the model (AUC > 0.70) but also provided a more precise performance estimate (with the width of CI reduced from 0.31 to 0.20). The optimism of the model was −0.03, indicating good robustness of the original model. (Supplementary Figure S3).

**Table 2:** Internal validation results of the random forest model using bootstrapping (B = 1,000).

Metric	Mean	95% CI
AUC	0.73	0.63–0.83
Accuracy	0.74	0.65–0.81
Recall	0.27	0.10–0.47
Specificity	0.91	0.81–0.98
PPV	0.55	0.27–0.86
NPV	0.77	0.67–0.86
F1 Score	0.35	0.15–0.54
ECE	0.11	0.06–0.18

Values are presented as Mean (95% Confidence Interval). CI, confidence interval; AUC, area under the receiver operating characteristic curve; PPV, positive predictive value; NPV, negative predictive value; ECE, expected calibration error.

### 3.3 Model description

**Figure 5:**
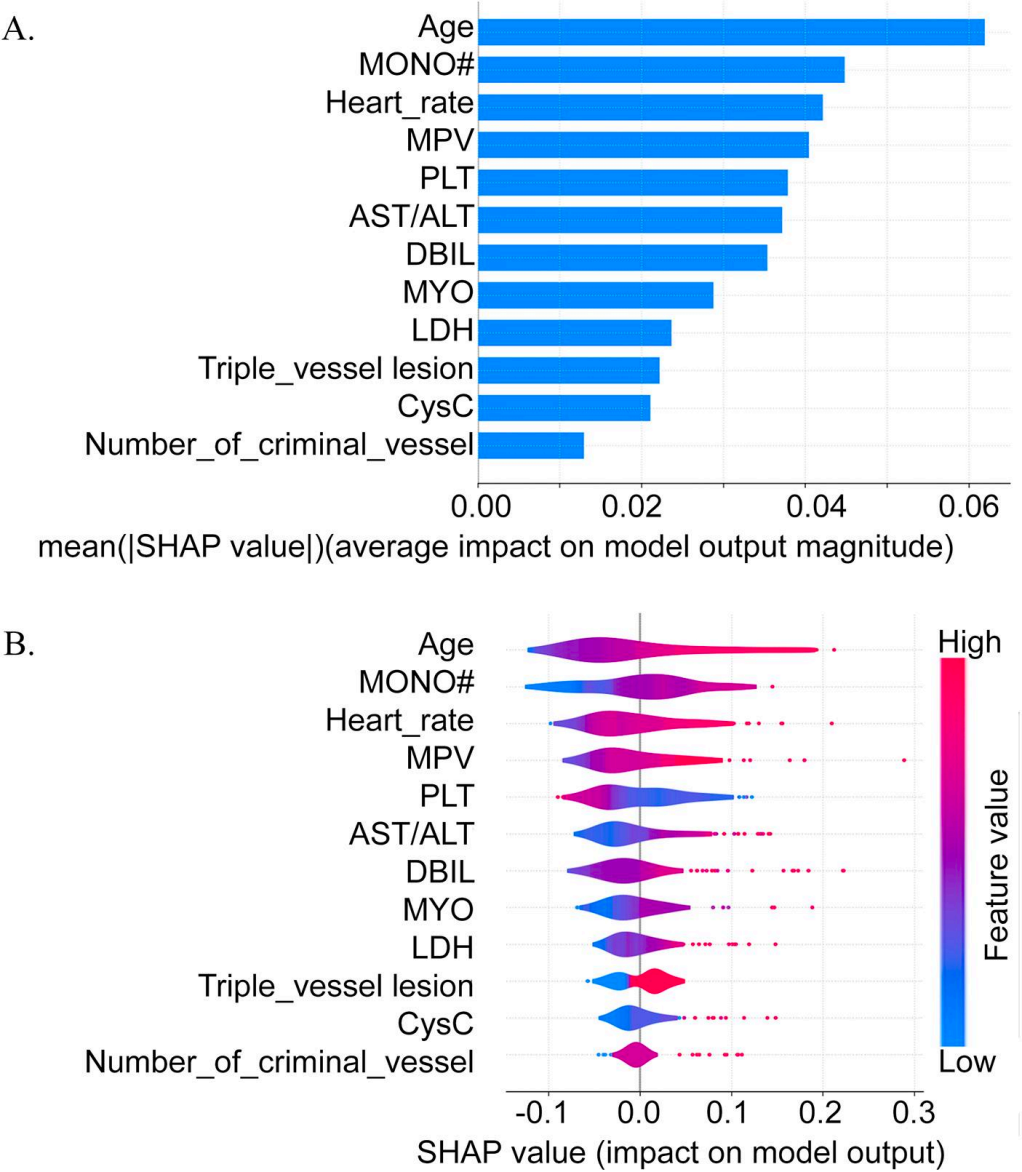
Summary SHAP plot of the top clinical features in the random forest model. **(A)**The average SHAP values for the 12 most important variables, which shows the typical extent of their impact on our model's predictions. Particularly important factors such as Age, Monocyte levels and Heart rate have the largest average SHAP values, indicating that they are often very important. **(B)**Distribution of SHAP values for each feature in all patient cases. Each dot represents a separate prediction, and the color represents the level of this feature (red is high, blue is low). This graph shows US how the magnitude and direction of a feature's influence change with its value; for example, higher Age always causes higher predictions, while conversely, lower PLT levels correspond to larger model output values.MONO#: monocyte count; MPV:mean platelet volume; PLT:platelet count; AST/ALT: aspartate aminotransferase to alanine aminotransferase ratio; DBIL:direct bilirubin; MYO: myoglobin; LDH: lactate dehydrogenase; CysC: cystatin.

**Figure 6:**
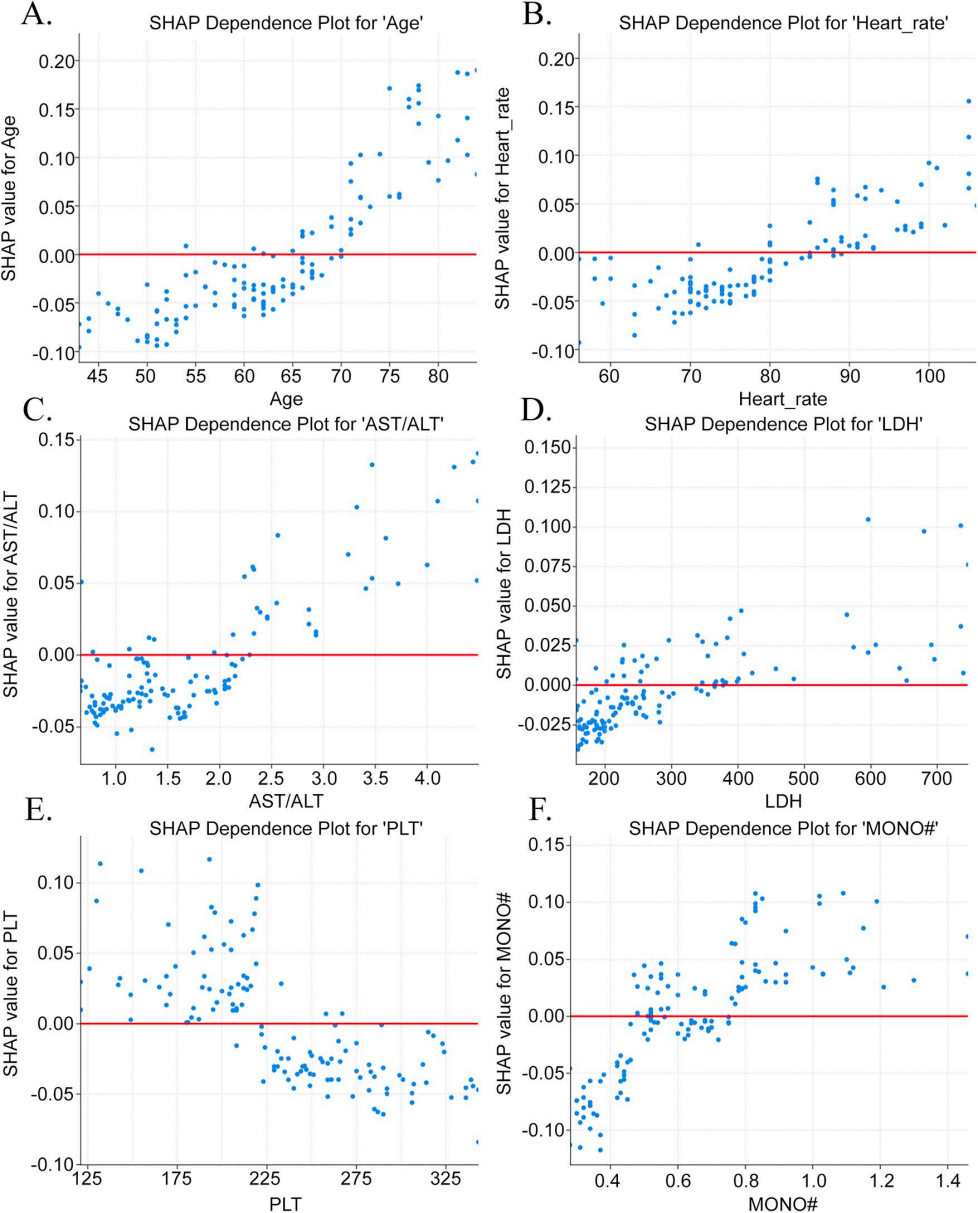
The relationship between critical clinical variables and the risk of in-hospital heart failure according to the random forest model. Subfigures **A** (Age), **B** (Heart rate), **C** (AST/ALT), **D** (LDH), and **F** (MONO#) all demonstrate a positive association with risk, where higher values correspond to increased risk. Conversely, Subfigures E (PLT) shows an inverse relationship, with lower platelet counts associated with higher risk.MONO#: monocyte count; PLT:platelet count; AST/ALT: aspartate aminotransferase to alanine aminotransferase ratio; LDH: lactate dehydrogenase.

The feature importance was assessed by SHAP values for the RF model on the validation cohort. The top 12 features based on the SHAP values (Figure 5A) and their positive (an increased risk) or negative (a decreased risk) contribution to the model's predictive efficacy (the SHAP summary plot in Figure 5B) were illustrated. The results revealed that the elevation of age, AST/ALT, cystatin C, myoglobin, number of diseased vessels, HR, absolute neutrophil count, direct bilirubin, LDH, and MPV, and the presence of triple vessel disease all made positive contributions, i.e., they increased the risk of HF. A decrease in PLT was also identified as a feature increasing the HF risk. To more deeply investigate how key features influence the probability of HF predicted by the RF model, a SHAP dependence plot was drawn for the six features (age, HR, AST/ALT, LDH, MONO#, and PLT) (Figure 6). The elevation of age, HR, AST/ALT, LDH, and MONO# was closely linked to an increased risk of HF, whereas the elevation of PLT was associated with a decreased risk.

### 3.4 Model application

**Figure 7:**
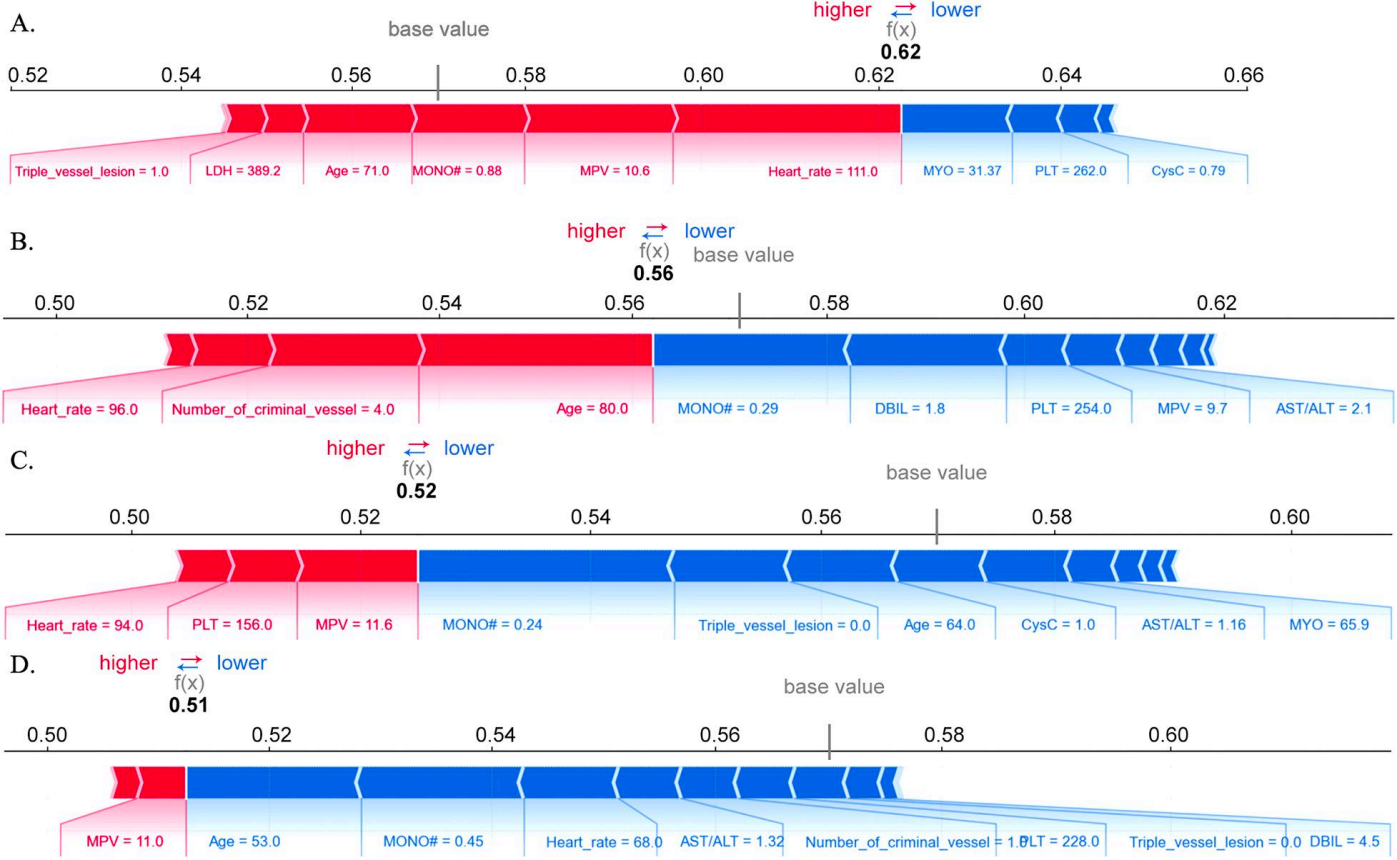
SHAP force analysis was applied to the random forest model to interpret the predictions for four patients from the validation set. The selected cases were individuals with acute ischemic heart disease post-PCI, including two who experienced in-hospital heart failure (sub-figures **A** and **B**) and two who did not (sub-figures **C** and **D**).MONO#: monocyte count; MPV:mean platelet volume; PLT:platelet count; AST/ALT: aspartate aminotransferase to alanine aminotransferase ratio; DBIL:direct bilirubin; MYO: myoglobin; LDH: lactate dehydrogenase; CysC: cystatin.

To further explore the application pattern of the RF model in specific patients, the HF risk was predicted for four patients (two HF and two non-HF) from the validation cohort, and visualized using the SHAP force plot (Figure 7). For the first HF patient, the predicted probability of HF was 0.62, and the main risk factors for HF were HR (111 bpm) and MPV (10.6 fL). For the second HF patient, the predicted probability of HF was 0.56, and the main risk factors were age (80 years) and the number of culprit vessels (4). For the two non-HF patients, the predicted probability of HF was 0.52 and 0.51, and the main protective factors were MONO (0.24 × 10^9^/L) and age (53 years), respectively.

**Figure 8:**
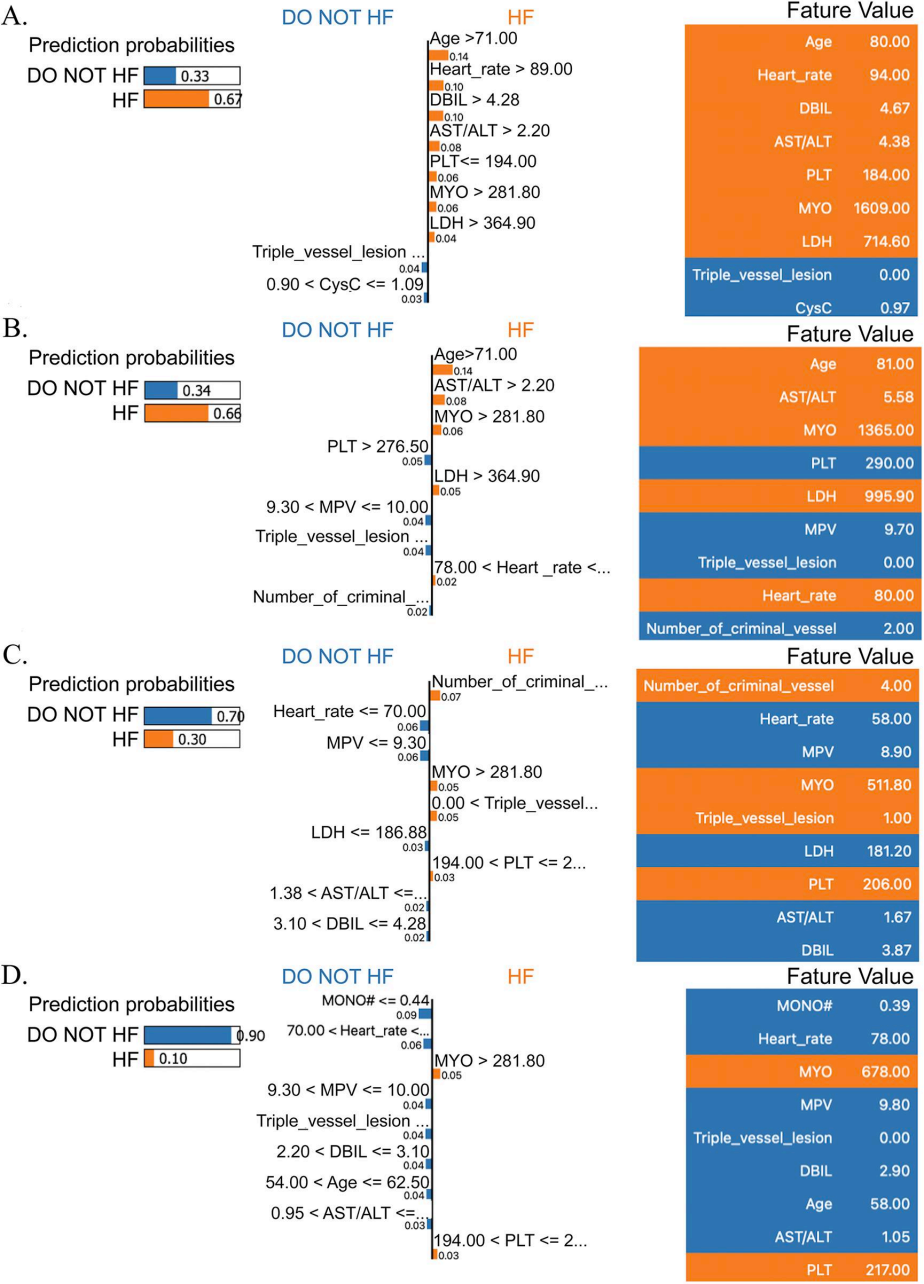
The LIME analysis findings for the random forest model focused on patients who had undergone PCI for acute ischemic heart disease. This included a pair of cases that developed in-hospital heart failure (shown in panels **A** and **B**) alongside two control subjects who remained free of this complication (depicted in panels **C** and **D**). MONO#: monocyte count; MPV:mean platelet volume; PLT:platelet count; AST/ALT: aspartate aminotransferase to alanine aminotransferase ratio; DBIL:direct bilirubin; MYO: myoglobin; LDH: lactate dehydrogenase; CysC: cystatin.

Additionally, the HF risk was also predicted for two patients by the LIME technique (Figure 8). For the first HF patient, the predicted probability of HF was 0.67, and the main risk factors for HF were age (81 years) and HR (94 bpm), while the protective factors were the absence of triple vessel disease and cystatin C (0.97 mg/L). For the second HF patient, the predicted probability of HF was 0.66, and the main risk factors for HF were age (81 years) and AST/ALT (5.58), while the protective factor was PLT (290 × 10^9^/L). For the two non-HF patients, the predicted probability of HF was 0.30 and 0.10, and the main protective factors were HR (58 bpm) and MONO (0.39 × 10^9^/L), respectively.

### 3.5 Web-based prediction tool

To promote the reproducibility and clinical application, the final prediction model has been deployed as an open-access Web-based tool freely accessible at https://hspifojdhapwpa7wwwadks.streamlit.app.

## 4 Discussion

In this study, 97 clinical features were collected from 203 AIHD patients within 24 h post-admission or during PCI, and assessed by LASSO regression and Pearson's coefficient of correlation. Finally, 12 key predictor variables were identified, based on which seven ML models were created for predicting the risk of post-PCI HF in AIHD and validated. The RF model exhibited optimal performance. In addition, the overall contribution of these features to the prediction of the RF model was analyzed by the SHAP technique, and the individualized prediction of the RF model for the HF risk was elucidated using the SHAP dependence plot and LIME technique, and an open-access Web-based prediction tool was deployed. The features incorporated in the model were all available through routine testing on admission, indicating the model's good clinical generalizability. The results showed that age, MONO#, HR, PLT, and MPV were five key features for predicting the risk of post-PCI HF in patients with AIHD. First, age has been identified previously as an independent risk factor for HF, which is implicated in the pathology of HF via promoting cardiac aging, altering the myocardial energy metabolism mode, and modulating the immuno-inflammatory response (20–22). In this study, age ranked first in the feature importance in the RF model, further suggesting that age is a core predictor of the risk of post-PCI HF in patients with AIHD. Second, tachycardia can directly contribute to myocardial remodeling by activating inflammatory and fibrotic pathways. In a tachycardia-induced HF model, the elevation of HR can significantly up-regulate TGF-*β* and MAPK signaling pathways as well as matrix metalloproteinases, thereby worsening extracellular matrix remodeling and myocardial fibrosis, which are key structural alterations in the HF development (23). Third, the relationship of platelet function with the HF prognosis remains controversial, but most studies suggest that platelet dysfunction often occurs in patients with HF. Patients with acute HF often present with marked platelet hyperactivation, underscoring an important role of platelets in the HF pathogenesis (24). Fourth, MONO is also identified as a predictor of HF, and monocytes and their cell subsets drive the progression of HF by mediating inflammatory responses and promoting myocardial fibrosis and hypertrophy (25). In addition, the elevation of MPV is positively associated with the HF risk according to available evidence (26).

HF is a common post-PCI complication in patients with AIHD and is closely linked to a significantly higher mortality (27). As shown by previous studies, ML techniques have been widely applied to clinical diagnosis and prognostic prediction, which can effectively enhance the accuracy of diagnosis and achieve individualized treatment (28, 29). For example, Lin et al. predicted the risk of HF in patients with AMI within three years post-PCI using RF, XGBoost, SVM, and LR models, with 45 variables incorporated, and found that left ventricular ejection fraction, left ventricular end-diastolic dimension, and LDH were the top three variables with the best predictive efficacy in the XGBoost model (14). However, all the above studies covered insufficient clinical features, and laboratory indicators, medication history, echocardiography and coronary angiography findings were included in only a few studies. Therefore, a predictive model integrating multidimensional clinical features is urgently needed to achieve a comprehensive assessment of the risk of post-PCI HF in patients with AIHD.

The advantages of this study are as follows: The model established focused on the prediction of the in-hospital HF, thereby achieving early risk warning and prompt intervention, and effectively reducing the in-hospital all-cause mortality and major adverse cardiovascular events. Moreover, 97 features were incorporated in the model, covering demographics, comorbidities, laboratory indicators, and coronary angiography findings. In particular, quantitative assessment was conducted on coronary angiography findings, including the degree of stenosis in each coronary artery segment, the number of culprit vessels, and the length and number of stents implanted. All variables were acquired from routine tests on admission or during PCI, greatly enhancing the clinical generalizability of the model. In addition, seven ML algorithms were systematically applied to the prediction of the risk of post-PCI in-hospital HF in AIHD patients. The results showed that the RF model exhibited optimal predictive performance: AUC 0.70 (95% CI 0.53–0.84), ECE 0.08, recall 0.33, F1-score 0.42, accuracy 0.77, PPV 0.56, and NPV 0.81. RF is an ensemble learning-based ML algorithm, and its excellent predictive performance and robustness have been verified (30, 31), especially in high-dimensional data processing and feature importance analysis (32). To enhance the interpretability, the prediction mechanism of the RF model was clarified by SHAP and LIME techniques. With improved model interpretability, clinicians can intuitively understand the prediction logic, enhancing the model's clinical utility. Another strength of this study is the easy-to-use digital interface developed. To ensure the practical applicability of our findings, an interactive Web-based risk calculator was constructed. This tool enables clinicians to input individual patient parameters and obtain immediate risk probabilities, thereby facilitating personalized medical decision-making.

However, this study also has several limitations. First, as a single-center retrospective cohort study with strict inclusion and exclusion criteria, the final sample size is 203 patients, involving 55 HF events. Although the variable-to-event ratio is slightly below the ideal threshold recommended by some statistical guidelines, we fully acknowledge this potential limitation in statistical power. To address this limitation, a robust Bootstrap internal validation method is specifically employed (1,000 Bootstrap iterations) to assess the robustness of the model with a limited sample size. The validation demonstrates an average AUC of 0.73 (95% CI: 0.63–0.83) for the adjusted model, with a moderately wide confidence interval, indicating reliable discriminative stability of the model under the current data structure. This exploratory study provides preliminary predictive evidence for identifying high-risk patients. Its external applicability and clinical generalizability remain to be further validated in future multicenter, large-scale prospective studies. Second, numerous clinical factors are not included in this study, such as LVEF, thrombolysis in myocardial infarction (TIMI) grade, medication details, and whether complementary and alternative medicine (CAM) interventions (including Chinese herbal medicine/acupuncture) are used alongside standardized drug therapy. These unassessed confounding factors may reduce the prediction accuracy of the RF model. Finally, the primary endpoint of this study is defined as in-hospital HF, excluding more objective hard endpoints like all-cause mortality from the main analysis. This limitation primarily stems from the extremely low number of in-hospital mortality events (4/203) in the current single-center cohort, resulting in insufficient statistical power for meaningful analysis. Future studies with longer follow-up periods are needed to accumulate sufficient endpoint events, thereby validating the value of this model in predicting composite hard endpoints including mortality.

## 5 Conclusion

The RF model demonstrates optimal performance in predicting the risk of post-PCI in-hospital HF in patients with AIHD. The model's clinical interpretability is greatly improved by SHAP and LIME techniques, enabling clinicians to intuitively understand the prediction logic and facilitating the model's clinical translation. In the future, multicenter prospective cohort studies can be conducted to validate the model's predictive efficacy and optimize the prediction algorithm in a diverse population.

## Data Availability

The original contributions presented in the study are included in the article/Supplementary Material, further inquiries can be directed to the corresponding author/s.

## Supplementary material

The Supplementary Materialfor this article can be found online at: https://www.frontiersin.org/articles/10.3389/fcvm.2026.1785285/full#supplementary-material

## References

[1] Byrne RA Rossello X Coughlan JJ Barbato E Berry C Chieffo A 2023 ESC guidelines for the management of acute coronary syndromes. Eur Heart J. (2023) 44(38):3720–826. 10.1093/eurheartj/ehad191 Erratum in: Eur Heart J. 2024 April 1;45(13):1145. doi: 10.1093/eurheartj/ehad870. PMID: 37622654. 37622654

[2] Nakamaru R Shiraishi Y Niimi N Ueda I Ikemura N Suzuki M Time trend in incidence of sudden cardiac death after percutaneous coronary intervention from 2009 to 2017 (from the Japanese multicenter registry). Am J Cardiol. (2023) 188:44–51. 10.1016/j.amjcard.2022.11.019 36470011

[3] Steg PG Dabbous OH Feldman LJ Cohen-Solal A Aumont MC López-Sendón J Global registry of acute coronary events investigators. Determinants and prognostic impact of heart failure complicating acute coronary syndromes: observations from the global registry of acute coronary events (GRACE). Circulation. (2004) 109(4):494–9. 10.1161/01.CIR.0000109691.16944.DA 14744970

[4] Li J Li X Wang Q Hu S Wang Y Masoudi FA ST-segment elevation myocardial infarction in China from 2001 to 2011(the China PEACE-retrospective acute myocardial infarction study):aretrospective analysis of hospital data. Lancet. (2015) 385(9966):441–51. 10.1016/S0140-6736(14)60921-1 24969506 PMC4415374

[5] Taniguchi T Shiomi H Morimoto T Watanabe H Ono K Shizuta S Incidence and prognostic impact of heart failure hospitalization during follow-up after primary percutaneous coronary intervention in ST-segment elevation myocardial infarction. Am J Cardiol. (2017) 119(11):1729–39. 10.1016/j.amjcard.2017.03.013 28407886

[6] Hu Y Dai S Chen L Ma X Li H Lu Y Multi-omics reveals the mechanism of vagus nerve stimulation in the treatment of chronic congestive heart failure. Sci Rep. (2025) 15(1):19613. 10.1038/s41598-025-04397-3 40467772 PMC12137920

[7] Xie H Wang Y Zhu X Zhang L Niu H Jin H. Revealing lactylation-mediated mechanisms and hub genes in heart failure pathogenesis. Front Cardiovasc Med. (2025) 12:1622958. 10.3389/fcvm.2025.1622958 40873619 PMC12378386

[8] Wu H Xia L Liu C. Apoptosis-Related non-coding RNAs in cardiac fibrosis and heart failure: implications for pathogenesis and therapy. J Inflamm Res. (2025) 18:11217–44. 10.2147/JIR.S541159 40860942 PMC12372813

[9] Wellings J Kostis JB Sargsyan D Cabrera J Kostis WJ. Myocardial infarction data acquisition system (MIDAS 31) study group. Risk factors and trends in incidence of heart failure following acute myocardial infarction. Am J Cardiol. (2018) 122(1):1–5. 10.1016/j.amjcard.2018.03.005 29685572

[10] Gerber Y Weston SA Enriquez-Sarano M Berardi C Chamberlain AM Manemann SM Mortality associated with heart failure after myocardial infarction: a contemporary community perspective. Circ Heart Fail. (2016) 9(1):e002460. 10.1161/CIRCHEARTFAILURE.115.002460 26699392 PMC4692179

[11] Laurent E Godillon L Tassi MF Marcollet P Chassaing S Decomis M Impact of cardiac rehabilitation and treatment compliance after ST-segment elevation myocardial infarction (STEMI) in France, the STOP SCA+ study. Front Cardiovasc Med. (2025) 12:1484401. 10.3389/fcvm.2025.1484401 40574816 PMC12198249

[12] Bu Z Bai S Yang C Lu G Lei E Su Y Application of an interpretable machine learning method to predict the risk of death during hospitalization in patients with acute myocardial infarction combined with diabetes mellitus. Acta Cardiol. (2025) 80(4):358–75. 10.1080/00015385.2025.2481662 40195951

[13] Ma Y Li M Wu H. The machine learning models in Major cardiovascular adverse events prediction based on coronary computed tomography angiography. Systematic Review. J Med Internet Res. (2025) 27:e68872. 10.2196/68872 40513092 PMC12205263

[14] Lin Q Zhao W Zhang H Chen W Lian S Ruan Q Predicting the risk of heart failure after acute myocardial infarction using an interpretable machine learning model. Front Cardiovasc Med. (2025) 12:1444323. 10.3389/fcvm.2025.1444323 39925976 PMC11802525

[15] Lundberg SM Erion G Chen H DeGrave A Prutkin JM Nair B From local explanations to global understanding with explainable AI for trees. Nat Mach Intell. (2020) 2(1):56–67. 10.1038/s42256-019-0138-9 32607472 PMC7326367

[16] Rudin C. Stop explaining black box machine learning models for high stakes decisions and use interpretable models instead. Nat Mach Intell. (2019) 1(5):206–15. 10.1038/s42256-019-0048-x 35603010 PMC9122117

[17] Riley RD Ensor J Snell KIE Harrell FE Jr Martin GP Reitsma JB Calculating the sample size required for developing a clinical prediction model. Br Med J. (2020) 368:m441. 10.1136/bmj.m441 32188600

[18] Collet JP Thiele H Barbato E Barthélémy O Bauersachs J Bhatt DL 2020 ESC guidelines for the management of acute coronary syndromes in patients presenting without persistent ST-segment elevation. Eur Heart J. (2021) 42(14):1289–367. 10.1093/eurheartj/ehaa575 Erratum in: Eur Heart J. 2021 May 14;42(19):1908. doi: 10.1093/eurheartj/ehaa895. Erratum in: Eur Heart J. 2021 May 14;42(19):1925. doi: 10.1093/eurheartj/ehab088. Erratum in: Eur Heart J. 2021 Jun 14;42(23):2298. doi: 10.1093/eurheartj/ehab285. Erratum in: Eur Heart J. 2024 Feb 1;45(5):404-405. doi: 10.1093/eurheartj/ehad879. PMID: 32860058. 32860058

[19] Chinese Society of Cardiology of Chinese Medical Association; Editorial Board of Chinese Journal of Cardiology. 2019 Chinese society of cardiology (CSC) guidelines for the diagnosis and management of patients with ST-segment elevation myocardial infarction. Zhonghua Xin Xue Guan Bing Za Zhi. (2019) 47(10):766–83. (Chinese). 10.3760/cma.j.issn.0253-3758.2019.10.003 31648459

[20] Zhou J Li Y Zhu L Yue R. Age differences in the association of prognostic nutritional index quartiles and heart failure in US adults: the NHANES 2011–2018. Front Nutr. (2025) 12:1533632. 10.3389/fnut.2025.1533632 40630165 PMC12234339

[21] Pun R Haas AL Thapa A Takafuji SR Suzuki RM Kay GF Bubr1 insufficiency drives transcriptomic alterations and pathology associated with cardiac aging and heart failure. Aging Cell. (2025) 24(9):e70160. 10.1111/acel.70160 40607964 PMC12419853

[22] Wang M Li H Han C Chen Y He L Xie M Loss of Elmsan1 in cardiomyocytes leads to age-dependent cardiac dysfunction and reduced lifespan. Am J Physiol Heart Circ Physiol. (2025) 329(2):H315–29. 10.1152/ajpheart.00810.2024 40588350 PMC12435183

[23] Plavelil N Goldstein R Klein MG Michaelson L Haigney MC Hood MN. Furosemide promotes inflammatory activation and myocardial fibrosis in swine with tachycardia-induced heart failure. Int J Mol Sci. (2025) 26(13):6088. 10.3390/ijms26136088 40649868 PMC12250366

[24] O'Connor CM Gurbel PA Serebruany VL. Usefulness of soluble and surface-bound P-selectin in detecting heightened platelet activity in patients with congestive heart failure. Am J Cardiol. (1999) 83(9):1345–9. 10.1016/s0002-9149(99)00098-3 10235093

[25] Li G Zhang C Li Y Yang J Wu J Shao Y Optogenetic vagal nerve stimulation attenuates heart failure by limiting the generation of monocyte-derived inflammatory CCRL2 + macrophages. Immunity. (2025) 58(7):1847–1861.e9. 10.1016/j.immuni.2025.06.003 40580954

[26] Wang B Wang J Liu C Hu X. The potential of platelet to high-density lipoprotein cholesterol ratio (PHR) as a novel biomarker for heart failure. Sci Rep. (2024) 14(1):23283. 10.1038/s41598-024-75453-7 39375501 PMC11458566

[27] Houdmont M Lim EH Djohan Hartanto A Lau V Chan SP Win SK Pathological Q waves at presentation of anterior ST segment elevation myocardial infarction predict heart failure: a southeast Asian perspective. Coron Artery Dis. (2025) 36(5):378–83. 10.1097/MCA.0000000000001475 39692434 PMC12199798

[28] Bu ZJ Jiang N Li KC Lu ZL Zhang N Yan SS Development and validation of an interpretable machine learning model for early prognosis prediction in ICU patients with malignant tumors and hyperkalemia. Medicine (Baltimore). (2024) 103(30):e38747. 10.1097/MD.0000000000038747 39058887 PMC11272258

[29] Foti G Spoto F Spezia A Romano L Caia S Camerani F Deep learning-driven abbreviated knee MRI protocols: diagnostic accuracy in clinical practice. Radiol Med. (2025) 130(9):1460–71. 10.1007/s11547-025-02038-3 40613973

[30] Ubels J Schaefers T Punt C Guchelaar HJ de Ridder J. RAINFOREST: a random forest approach to predict treatment benefit in data from (failed) clinical drug trials. Bioinformatics. (2020) 36(Suppl_2):i601–9. 10.1093/bioinformatics/btaa799 33381829

[31] Zhang X Huang Y Wang Y Jiang Y Liu B Ren J Development and external validation of nomogram associated with gastroparesis syndrome after subtotal gastrectomy depending on random forest and traditional model: does robotic surgery have advantages? J Robot Surg. (2025) 19(1):167. 10.1007/s11701-025-02259-8 40257665

[32] Akbar SB Thanupillai K Sundararaj S. Combining the advantages of AlexNet convolutional deep neural network optimized with anopheles search algorithm based feature extraction and random forest classifier for COVID-19 classification. Concurr Comput. (2022) 34(15):e6958. 10.1002/cpe.6958 35573661 PMC9087014

