# Supplementary material for: Establishment and validation of an interpretable machine learning-based predictive model for risk of post-PCI in-hospital heart failure in AIHD patients
Source: Front Cardiovasc Med. 2026 Feb 27;13:1785285. doi: 10.3389/fcvm.2026.1785285 (PMC12982109; doi:10.3389/fcvm.2026.1785285)
Supplement: Supplementary file 1 [file Datasheet1.doc]

Supplementary Material

Establishment and Validation of an Interpretable Machine Learning-Based Predictive Model for Risk of Post-PCI In-Hospital Heart Failure in AIHD Patients

Xinying Zhao[[1]](#footnote-2),Zhihang Wang^1^,Qiqi Yang^1^,Huiqi Liu^2^,Yigen Li^3^,Xi Ye^1^*

^1^The Affiliated Guangzhou Hospital of TCM of Guangzhou University of Chinese Medicine, Guangzhou Guangdong 510130, China

^2^School of Computer Science, University of Bristol, Bristol BS8 1UB, United Kingdom

**^3^**National TCM Master Lin Tiandong’s Heritage and Inheritance Studio, The Affiliated Guangzhou Hospital of TCM of Guangzhou University of Chinese Medicine, Guangzhou Guangdong 510130, China

*** Correspondence:**Xi Ye
yexi_1039@163.com

Supplementary Tables and Figures cited in main text

• Table S1.All extracted variables in the hospital database (N=118)

• Table S2.Missing data summary for all variables stratified by outcome.

• Table S3: Clinical features used for developing the models (N=97).

• Table S4.The rankings of each model.

• Table S5.Detailed specifications and hyperparameter settings for the machine learning algorithms.

• Figure S1.Feature selection using the LASSO regression model.

• Figure S2.Pearson correlation heatmap of the selected clinical features.

• Figure S3.Distribution of Area Under the Curve (AUC) scores from Bootstrap internal validation of the Random Forest model.

| **Table S1.** All extracted variables in the hospital database (N=118). | |
| --- | --- |
| Gender | Demographic characteristics (2) |
| Age |
| Smoking | Vital signs (6) |
| Alcohol |
| Heart rate |
| Systolic blood pressure |
| Diastolic blood pressure |
| Hypertension | Medical history (5) |
| Diabetes |
| Coronary heart disease |
| Number of stent（before） |
| Hyperlipidemia |
| STEMI | Classification of illness（1） |
| LM | Coronary angiogram findings（15） |
| LAD |
| pLAD |
| mLAD |
| dLAD |
| LCX |
| pLCX |
| dLCX |
| RCA |
| pRCA |
| mRCA |
| dRCA |
| Triple vessel lesion |
| Main criminal vessel |
| Number of criminal vessel |
| Number of stents | Coronary stent（2） |
| Length of stents |
| Serum potassium | Laboratory findings (88) |
| Serum sodium |
| Serum chloride |
| Serum bicarbonate |
| Serum calcium |
| Serum magnesium |
| Serum phosphate |
| Alanine Aminotransferase |
| Aspartate Aminotransferase |
| Aspartate Aminotransferase to Alanine Aminotransferase Ratio |
| Gamma-Glutamyl Transferase |
| Alkaline Phosphatase |
| Glutathione Reductase |
| α-L-Fucosidase |
| Total Protein |
| Albumin |
| Globulin |
| Albumin to Globulin Ratio |
| Prealbumin |
| Total Bilirubin |
| Direct Bilirubin |
| Indirect Bilirubin |
| Total Bile Acids |
| Glucose |
| Uric Acid |
| Blood Urea Nitrogen |
| Creatinine |
| Cystatin C |
| Retinol-Binding Protein |
| Lactate Dehydrogenase |
| Creatine Kinase |
| Creatine Kinase-Myocardial Band |
| Hydroxybutyrate Dehydrogenase |
| Total Cholesterol |
| Triglycerides |
| High-Density Lipoprotein Cholesterol |
| Low-Density Lipoprotein Cholesterol |
| Prothrombin Time |
| Prothrombin Time Percentage |
| Prothrombin Time Ratio |
| International Normalized Ratio |
| Activated Partial Thromboplastin Time |
| D-Dimer |
| White Blood Cell Count |
| Neutrophil Count (Absolute) |
| Neutrophil Percentage |
| Lymphocyte Count (Absolute) |
| Lymphocyte Percentage |
| Monocyte Count (Absolute) |
| Monocyte Percentage |
| Eosinophil Count (Absolute) |
| Eosinophil Percentage |
| Basophil Count (Absolute) |
| Basophil Percentage |
| Red Blood Cell Count |
| Hemoglobin |
| Hematocrit |
| Mean Corpuscular Volume |
| Mean Corpuscular Hemoglobin |
| Mean Corpuscular Hemoglobin Concentration |
| Red Cell Distribution Width - Coefficient of Variation |
| Red Cell Distribution Width - Standard Deviation |
| Platelet Count |
| Mean Platelet Volume |
| Plateletcrit |
| Platelet Distribution Width |
| High-Sensitivity Cardiac Troponin T |
| Myoglobin |
| N-Terminal pro-B-type Natriuretic Peptide |
| Arterial plasma potassium |
| Arterial plasma Sodium |
| Arterial plasma Chloride |
| Arterial plasma Ionized Calcium |
| Arterial plasma Glucose |
| pH |
| Partial Pressure of Carbon Dioxide |
| Partial Pressure of Oxygen |
| Oxygen Saturation |
| Hematocrit |
| Base Excess of Extracellular Fluid |
| Base Excess of Blood |
| Standard Bicarbonate Concentration |
| Bicarbonate Ion |
| Total Carbon Dioxide Content |
| Alveolar Oxygen Partial Pressure |
| Alveolar-arterial Oxygen Gradient |
| a/A Ratio |
| Lactate |

| **Table S2.**Missing data summary for all variables stratified by outcome. | | | |
| --- | --- | --- | --- |
| Variable Name | Overall Missing n (%)  (N=203) | Non-HF Missing n (%)  (n=148) | HF Missing n (%) (n=55) |
| Gender | 0 (0.0%) | 0 (0.0%) | 0 (0.0%) |
| Age | 0 (0.0%) | 0 (0.0%) | 0 (0.0%) |
| Hypertension | 0 (0.0%) | 0 (0.0%) | 0 (0.0%) |
| Diabetes | 0 (0.0%) | 0 (0.0%) | 0 (0.0%) |
| coronary heart disease | 0 (0.0%) | 0 (0.0%) | 0 (0.0%) |
| Hyperlipidemia | 0 (0.0%) | 0 (0.0%) | 0 (0.0%) |
| Smoking | 0 (0.0%) | 0 (0.0%) | 0 (0.0%) |
| alcohol | 0 (0.0%) | 0 (0.0%) | 0 (0.0%) |
| Heart rate | 13 (6.4%) | 5 (3.4%) | 8 (14.5%) |
| Systolic blood pressure | 14 (6.9%) | 6 (4.1%) | 8 (14.5%) |
| Diastolic blood pressure | 14 (6.9%) | 6 (4.1%) | 8 (14.5%) |
| STEMI | 0 (0.0%) | 0 (0.0%) | 0 (0.0%) |
| Number of stent（before） | 2 (1.0%) | 2 (1.4%) | 0 (0.0%) |
| LM | 3 (1.5%) | 2 (1.4%) | 1 (1.8%) |
| LAD | 3 (1.5%) | 2 (1.4%) | 1 (1.8%) |
| pLAD | 3 (1.5%) | 2 (1.4%) | 1 (1.8%) |
| mLAD | 30 (14.8%) | 15 (10.1%) | 15 (27.3%) |
| dLAD | 40 (19.7%) | 21 (14.2%) | 19 (34.5%) |
| LCX | 3 (1.5%) | 2 (1.4%) | 1 (1.8%) |
| pLCX | 3 (1.5%) | 2 (1.4%) | 1 (1.8%) |
| dLCX | 8 (3.9%) | 5 (3.4%) | 3 (5.5%) |
| RCA | 3 (1.5%) | 2 (1.4%) | 1 (1.8%) |
| pRCA | 3 (1.5%) | 2 (1.4%) | 1 (1.8%) |
| mRCA | 20 (9.9%) | 15 (10.1%) | 5 (9.1%) |
| dRCA | 24 (11.8%) | 19 (12.8%) | 5 (9.1%) |
| Number of criminal vessel | 0 (0.0%) | 0 (0.0%) | 0 (0.0%) |
| Triple vessel lesion | 3 (1.5%) | 2 (1.4%) | 1 (1.8%) |
| Main criminal vessel | 16 (7.9%) | 12 (8.1%) | 4 (7.3%) |
| Number of stents | 3 (1.5%) | 2 (1.4%) | 1 (1.8%) |
| Length of stents | 3 (1.5%) | 2 (1.4%) | 1 (1.8%) |
| Serum potassium | 16 (7.9%) | 13 (8.8%) | 3 (5.5%) |
| Serum sodium | 18 (8.9%) | 14 (9.5%) | 4 (7.3%) |
| Serum chloride | 16 (7.9%) | 14 (9.5%) | 2 (3.6%) |
| Serum bicarbonate | 37 (18.2%) | 28 (18.9%) | 9 (16.4%) |
| Serum calcium | 33 (16.3%) | 25 (16.9%) | 8 (14.5%) |
| Serum magnesium | 33 (16.3%) | 24 (16.2%) | 9 (16.4%) |
| Serum phosphate | 39 (19.2%) | 29 (19.6%) | 10 (18.2%) |
| Alanine Aminotransferase | 24 (11.8%) | 18 (12.2%) | 6 (10.9%) |
| Aspartate Aminotransferase | 14 (6.9%) | 11 (7.4%) | 3 (5.5%) |
| Aspartate Aminotransferase to Alanine Aminotransferase Ratio | 30 (14.8%) | 23 (15.5%) | 7 (12.7%) |
| Gamma-Glutamyl Transferase | 28 (13.8%) | 19 (12.8%) | 9 (16.4%) |
| Alkaline Phosphatase | 38 (18.7%) | 30 (20.3%) | 8 (14.5%) |
| Glutathione Reductase | 39 (19.2%) | 30 (20.3%) | 9 (16.4%) |
| α-L-Fucosidase | 37 (18.2%) | 29 (19.6%) | 8 (14.5%) |
| Total Protein | 32 (15.8%) | 27 (18.2%) | 5 (9.1%) |
| Albumin | 34 (16.7%) | 28 (18.9%) | 6 (10.9%) |
| Globulin | 33 (16.3%) | 27 (18.2%) | 6 (10.9%) |
| Albumin to Globulin Ratio | 35 (17.2%) | 29 (19.6%) | 6 (10.9%) |
| Prealbumin | 43 (21.2%) | 32 (21.6%) | 11 (20.0%) |
| Total Bilirubin | 27 (13.3%) | 22 (14.9%) | 5 (9.1%) |
| Direct Bilirubin | 28 (13.8%) | 24 (16.2%) | 4 (7.3%) |
| Indirect Bilirubin | 29 (14.3%) | 24 (16.2%) | 5 (9.1%) |
| Total Bile Acids | 35 (17.2%) | 26 (17.6%) | 9 (16.4%) |
| Glucose | 31 (15.3%) | 27 (18.2%) | 4 (7.3%) |
| Uric Acid | 28 (13.8%) | 24 (16.2%) | 4 (7.3%) |
| Blood Urea Nitrogen | 28 (13.8%) | 23 (15.5%) | 5 (9.1%) |
| Creatinine | 23 (11.3%) | 18 (12.2%) | 5 (9.1%) |
| Cystatin C | 51 (25.1%) | 40 (27.0%) | 11 (20.0%) |
| Retinol-Binding Protein | 74 (36.5%) | 59 (39.9%) | 15 (27.3%) |
| Lactate Dehydrogenase | 19 (9.4%) | 16 (10.8%) | 3 (5.5%) |
| Creatine Kinase | 16 (7.9%) | 13 (8.8%) | 3 (5.5%) |
| Creatine Kinase-Myocardial Band | 13 (6.4%) | 12 (8.1%) | 1 (1.8%) |
| Hydroxybutyrate Dehydrogenase | 18 (8.9%) | 16 (10.8%) | 2 (3.6%) |
| Total Cholesterol | 42 (20.7%) | 29 (19.6%) | 13 (23.6%) |
| Triglycerides | 42 (20.7%) | 29 (19.6%) | 13 (23.6%) |
| High-Density Lipoprotein Cholesterol | 42 (20.7%) | 30 (20.3%) | 12 (21.8%) |
| Low-Density Lipoprotein Cholesterol | 40 (19.7%) | 27 (18.2%) | 13 (23.6%) |
| Prothrombin Time | 24 (11.8%) | 17 (11.5%) | 7 (12.7%) |
| Prothrombin Time Percentage | 25 (12.3%) | 18 (12.2%) | 7 (12.7%) |
| Prothrombin Time Ratio | 23 (11.3%) | 16 (10.8%) | 7 (12.7%) |
| International Normalized Ratio | 24 (11.8%) | 17 (11.5%) | 7 (12.7%) |
| Activated Partial Thromboplastin Time | 25 (12.3%) | 18 (12.2%) | 7 (12.7%) |
| D-Dimer | 46 (22.7%) | 34 (23.0%) | 12 (21.8%) |
| White Blood Cell Count | 19 (9.4%) | 13 (8.8%) | 6 (10.9%) |
| Neutrophil Count (Absolute) | 18 (8.9%) | 13 (8.8%) | 5 (9.1%) |
| Neutrophil Percentage | 22 (10.8%) | 15 (10.1%) | 7 (12.7%) |
| Lymphocyte Count (Absolute) | 20 (9.9%) | 14 (9.5%) | 6 (10.9%) |
| Lymphocyte Percentage | 20 (9.9%) | 14 (9.5%) | 6 (10.9%) |
| Monocyte Count (Absolute) | 20 (9.9%) | 13 (8.8%) | 7 (12.7%) |
| Monocyte Percentage | 21 (10.3%) | 15 (10.1%) | 6 (10.9%) |
| Eosinophil Count (Absolute) | 18 (8.9%) | 13 (8.8%) | 5 (9.1%) |
| Eosinophil Percentage | 18 (8.9%) | 13 (8.8%) | 5 (9.1%) |
| Basophil Count (Absolute) | 19 (9.4%) | 14 (9.5%) | 5 (9.1%) |
| Basophil Percentage | 18 (8.9%) | 13 (8.8%) | 5 (9.1%) |
| Red Blood Cell Count | 20 (9.9%) | 14 (9.5%) | 6 (10.9%) |
| Hemoglobin | 20 (9.9%) | 14 (9.5%) | 6 (10.9%) |
| Hematocrit | 19 (9.4%) | 14 (9.5%) | 5 (9.1%) |
| Mean Corpuscular Volume | 20 (9.9%) | 15 (10.1%) | 5 (9.1%) |
| Mean Corpuscular Hemoglobin | 21 (10.3%) | 14 (9.5%) | 7 (12.7%) |
| Mean Corpuscular Hemoglobin Concentration | 20 (9.9%) | 14 (9.5%) | 6 (10.9%) |
| Red Cell Distribution Width - Coefficient of Variation | 21 (10.3%) | 14 (9.5%) | 7 (12.7%) |
| Red Cell Distribution Width - Standard Deviation | 22 (10.8%) | 15 (10.1%) | 7 (12.7%) |
| Platelet Count | 20 (9.9%) | 15 (10.1%) | 5 (9.1%) |
| Mean Platelet Volume | 24 (11.8%) | 18 (12.2%) | 6 (10.9%) |
| Plateletcrit | 25 (12.3%) | 18 (12.2%) | 7 (12.7%) |
| Platelet Distribution Width | 32 (15.8%) | 23 (15.5%) | 9 (16.4%) |
| High-Sensitivity Cardiac Troponin T | 60 (29.6%) | 44 (29.7%) | 16 (29.1%) |
| Myoglobin | 45 (22.2%) | 37 (25.0%) | 8 (14.5%) |
| N-Terminal pro-B-type Natriuretic Peptide | 18 (8.9%) | 15 (10.1%) | 3 (5.5%) |
| Arterial plasma potassium | 149 (73.4%) | 109 (73.6%) | 40 (72.7%) |
| Arterial plasma Sodium | 149 (73.4%) | 109 (73.6%) | 40 (72.7%) |
| Arterial plasma Chloride | 149 (73.4%) | 109 (73.6%) | 40 (72.7%) |
| Arterial plasma Ionized Calcium | 150 (73.9%) | 110 (74.3%) | 40 (72.7%) |
| Arterial plasma Glucose | 150 (73.9%) | 110 (74.3%) | 40 (72.7%) |
| pH | 150 (73.9%) | 109 (73.6%) | 41 (74.5%) |
| Partial Pressure of Carbon Dioxide | 150 (73.9%) | 109 (73.6%) | 41 (74.5%) |
| Partial Pressure of Oxygen | 150 (73.9%) | 109 (73.6%) | 41 (74.5%) |
| Oxygen Saturation | 150 (73.9%) | 109 (73.6%) | 41 (74.5%) |
| Hematocrit | 150 (73.9%) | 109 (73.6%) | 41 (74.5%) |
| Base Excess of Extracellular Fluid | 150 (73.9%) | 109 (73.6%) | 41 (74.5%) |
| Base Excess of Blood | 150 (73.9%) | 109 (73.6%) | 41 (74.5%) |
| Standard Bicarbonate Concentration | 150 (73.9%) | 109 (73.6%) | 41 (74.5%) |
| Bicarbonate Ion | 150 (73.9%) | 109 (73.6%) | 41 (74.5%) |
| Total Carbon Dioxide Content | 150 (73.9%) | 109 (73.6%) | 41 (74.5%) |
| Alveolar Oxygen Partial Pressure | 150 (73.9%) | 109 (73.6%) | 41 (74.5%) |
| Alveolar-arterial Oxygen Gradient | 166 (81.8%) | 121 (81.8%) | 45 (81.8%) |
| a/A Ratio | 150 (73.9%) | 109 (73.6%) | 41 (74.5%) |
| Lactate | 153 (75.4%) | 111 (75.0%) | 42 (76.4%) |
| Variables with above 30% missing values were removed from further analysis.a/A Ratio：The arterial-to-alveolar oxygen tension ratio | | | |

| **Table S3.** Clinical features used for developing the models (N=97). |  |
| --- | --- |
| Gender | Demographic characteristics (2) |
| Age |
| Smoking | Vital signs (6) |
| Heavy Drinking |
| Heart rate |
| Systolic blood pressure |
| Diastolic blood pressure |
| Hypertension | Medical history (5) |
| Diabetes |
| Coronary heart disease |
| Previously implanted stent |
| Hyperlipidemia |
| STEMI | Classification of illness（1） |
| LM | Coronary angiogram findings（15） |
| LAD |
| pLAD |
| mLAD |
| dLAD |
| LCX |
| pLCX |
| dLCX |
| RCA |
| pRCA |
| mRCA |
| dRCA |
| Triple vessel lesion |
| Main criminal vessel |
| Number of criminal vessel |
| Number of stents | Coronary stent（2） |
| Length of stents |
| Potassium | Laboratory findings (67) |
| Sodium |
| Chloride |
| Bicarbonate |
| Calcium |
| Magnesium |
| Phosphate |
| Alanine Aminotransferase |
| Aspartate Aminotransferase |
| Aspartate Aminotransferase to Alanine Aminotransferase Ratio |
| Gamma-Glutamyl Transferase |
| Alkaline Phosphatase |
| Glutathione Reductase |
| α-L-Fucosidase |
| Total Protein |
| Albumin |
| Globulin |
| Albumin to Globulin Ratio |
| Prealbumin |
| Total Bilirubin |
| Direct Bilirubin |
| Indirect Bilirubin |
| Total Bile Acids |
| Glucose |
| Uric Acid |
| Blood Urea Nitrogen |
| Creatinine |
| Cystatin C |
| Lactate Dehydrogenase |
| Creatine Kinase |
| Creatine Kinase-Myocardial Band |
| Hydroxybutyrate Dehydrogenase |
| Total Cholesterol |
| Triglycerides |
| High-Density Lipoprotein Cholesterol |
| Low-Density Lipoprotein Cholesterol |
| Prothrombin Time |
| Prothrombin Time Percentage |
| Prothrombin Time Ratio |
| International Normalized Ratio |
| Activated Partial Thromboplastin Time |
| D-Dimer |
| White Blood Cell Count |
| Neutrophil Count (Absolute) |
| Neutrophil Percentage |
| Lymphocyte Count (Absolute) |
| Lymphocyte Percentage |
| Monocyte Count (Absolute) |
| Monocyte Percentage |
| Eosinophil Count (Absolute) |
| Eosinophil Percentage |
| Basophil Count (Absolute) |
| Basophil Percentage |
| Red Blood Cell Count |
| Hematocrit |
| Mean Corpuscular Volume |
| Mean Corpuscular Hemoglobin |
| Mean Corpuscular Hemoglobin Concentration |
| Red Cell Distribution Width - Coefficient of Variation |
| Red Cell Distribution Width - Standard Deviation |
| Platelet Count |
| Mean Platelet Volume |
| Plateletcrit |
| Platelet Distribution Width |
| High-Sensitivity Cardiac Troponin T |
| Myoglobin |
| N-Terminal pro-B-type Natriuretic Peptide |

**Table S4.**Detailed specifications and hyperparameter settings for the machine learning algorithms.

| Algorithm | Implementation Library | Key Hyperparameters & Settings |
| --- | --- | --- |
| Logistic Regression | sklearn.linear model | Penalty: L2; Solver: lbfgs; C: 1.0 (Default) |
| SVM (RBF Kernel) | sklearn.svm | Kernel: RBF; Probability: True (Internal Platt Scaling enabled); C: 1.0; Gamma: scale |
| K-Nearest Neighbors | sklearn.neighbors | Number of neighbors (k): 3; Weights: uniform |
| Decision Tree | sklearn.tree | Criterion: Gini impurity; Splitter: Best; Max depth: None |
| Random Forest | sklearn.ensemble | Number of estimators: 100; Criterion: Gini; Random State: 0 |
| Naive Bayes | sklearn.naive bayes | Type: GaussianNB; Priors: None (Data-driven) |
| XGBoost | xgboost | Booster: gbtree; Learning rate: 0.3; Max depth: 6; Objective: binary:logistic |

This table outlines the machine learning algorithms and their corresponding implementation libraries used in this study. Key hyperparameters derived from sklearn (Scikit-Learn) and xgboost frameworks are listed. Unless otherwise specified, default parameters were used. For the SVM model, internal Platt Scaling was enabled (Probability: True) to allow for probability estimates. A fixed random state (where applicable, e.g., Random Forest) was used to ensure reproducibility.

**Table S5.**The rankings of each model.

| Model | AUC | AUC (95% CI) | ECE | Recall | F1 Score | Accuracy | PPV | NPV | Overall Score |
| --- | --- | --- | --- | --- | --- | --- | --- | --- | --- |
| Random Forest | 0.70 | 0.53–0.84 | 0.08 | 0.33 | 0.42 | 0.77 | 0.56 | 0.81 | 2.07 |
| K-Nearest Neighbors | 0.57 | 0.38–0.75 | 0.10 | 0.40 | 0.43 | 0.74 | 0.46 | 0.81 | 2.79 |
| Naive Bayes | 0.68 | 0.52–0.83 | 0.16 | 0.33 | 0.38 | 0.74 | 0.45 | 0.8 | 3.79 |
| XGBoost | 0.72 | 0.56–0.86 | 0.18 | 0.33 | 0.37 | 0.72 | 0.42 | 0.80 | 4.21 |
| SVM (RBF Kernel) | 0.62 | 0.43–0.80 | 0.14 | 0.20 | 0.32 | 0.79 | 0.75 | 0.79 | 4.36 |
| Decision Tree | 0.58 | 0.44–0.72 | 0.15 | 0.40 | 0.38 | 0.67 | 0.35 | 0.80 | 4.64 |
| Logistic Regression | 0.65 | 0.47–0.82 | 0.18 | 0.27 | 0.32 | 0.72 | 0.40 | 0.78 | 5.93 |
| Overall Score: Average of ranks for each metric (lower value is better for ECE). Lower Overall Scores denote better model performance. | | | | | | | | | |

**Figure S1.** Feature selection using the LASSO regression model.


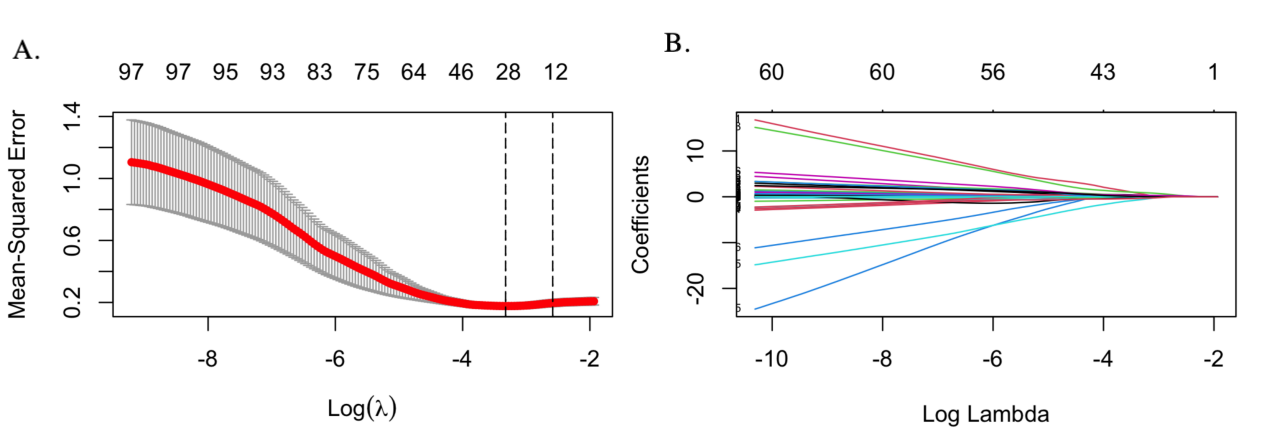


(A) LASSO coefficient patterns across 97 initial variables.

(B)LASSO model tuning parameter (λ) determination employed 5-fold cross-validation through minimum criteria.

LASSO：least absolute shrinkage and selection operator.

**Figure S2.**Pearson correlation heatmap of the selected clinical features.


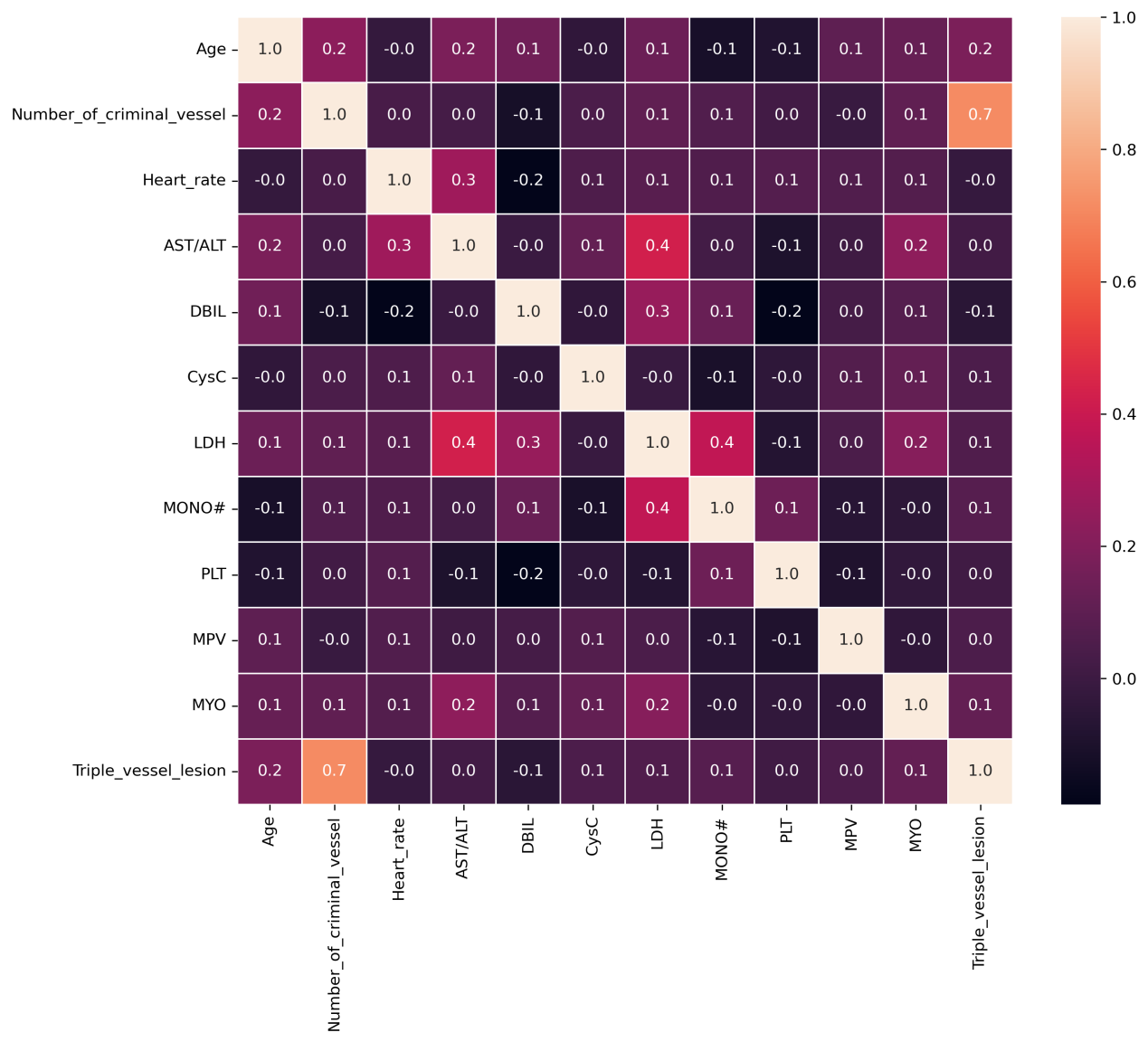


The heatmap illustrates the pairwise Pearson correlation coefficients among the 12 predictor variables used in the model. Variables include demographics, angiographic characteristics (e.g., Number of criminal vessels, Triple vessel lesion), and laboratory indices (e.g., AST/ALT, LDH, MYO). The numerical value in each cell represents the correlation coefficient. The color scale on the right indicates the strength of the correlation: lighter colors (orange) denote stronger positive correlations, while darker colors (purple/black) indicate weaker correlations. The dominance of darker shades suggests a lack of severe multicollinearity among the selected features.

**Figure S3.**Distribution of Area Under the Curve (AUC) scores from Bootstrap internal validation of the Random Forest model.


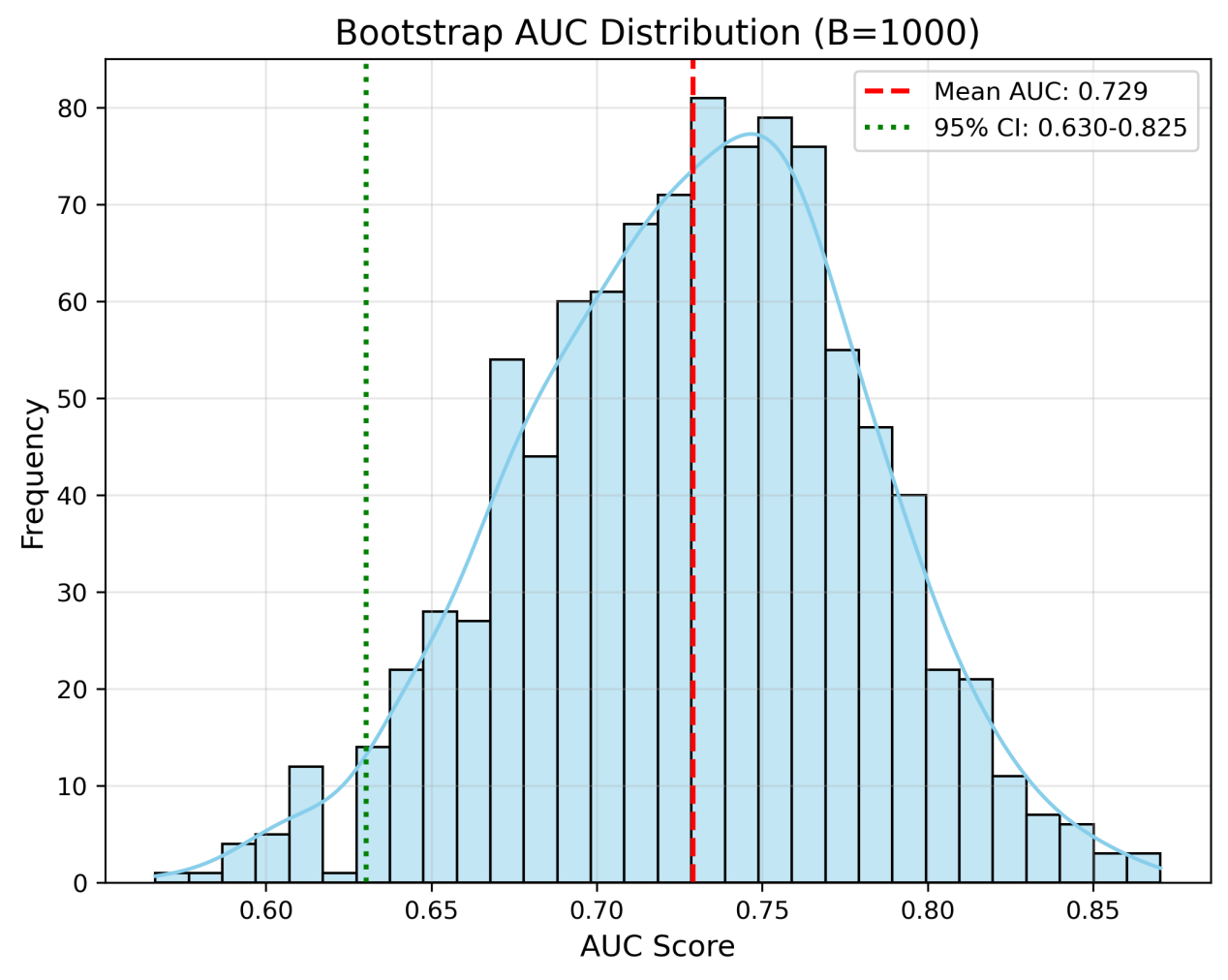


The histogram displays the frequency distribution of AUC scores obtained from B bootstrap iterations. The solid blue bars represent the frequency of specific AUC values, overlaid with a Kernel Density Estimation (KDE) curve. The red dashed line indicates the mean AUC value. The green dotted lines delineate the lower and upper bounds of the 95% confidence interval (95% CI). This visualization demonstrates the robustness and variability of the model's predictive performance.

1. [↑](#footnote-ref-2)
